# Supplementary material for: Design and Synthesis of D3R Bitopic Ligands with Flexible Secondary Binding Fragments: Radioligand Binding and Computational Chemistry Studies
Source: Molecules. 2023 Dec 24;29(1):123. doi: 10.3390/molecules29010123 (PMC10779535; doi:10.3390/molecules29010123)

Supplementary Materials

Design and Synthesis of D_3R Bitopic Ligands with Flexible Secondary Binding Fragments: Radioligand Binding and Computational Chemistry Studies

Gui-Long Tian [1], Chia-Ju Hsieh [1], Michelle Taylor [2], Ji Youn Lee [1], Robert R. Luedtke [2] and Robert H. Mach [1]

[1] Division of Nuclear Medicine and Clinical Molecular Imaging, Department of Radiology, Perelman School of Medicine, University of Pennsylvania, Philadelphia, PA 19104, USA
[2] Department of Pharmacology and Neuroscience, University of North Texas Health Science Center-Fort Worth, Texas, TX 76107, USA
* Correspondence: rmach@pennmedicine.upenn.edu

Compounds purity and graphs.

**Compounds**	Structure	Purity
**(R, S)-trans-2a**	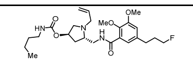	99.99
**(R, S)-trans-2b**	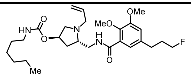	99.99
**(R, S)-trans-2c**	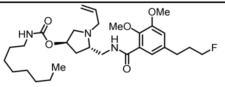	95.19
**(R, S)-trans-2d**	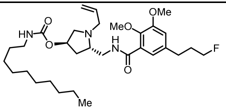	99.99
**(R, S)-trans-2e**	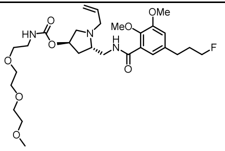	98.55
**(R, S)-trans-2f**	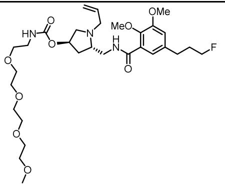	97.72
**(R, S)-trans-2g**	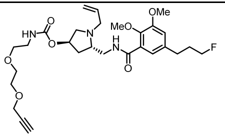	95.34
**(R, S)-trans-2h**	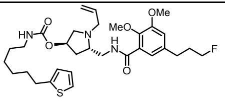	97.02
**(R, S)-trans-2i**	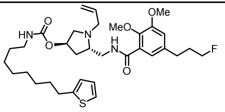	97.70
**(R, S)-trans-2j**	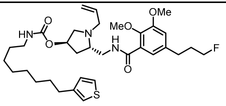	99.99
**(S, R)-trans-2d**	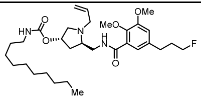	96.48
**(S, S)-cis-2d**	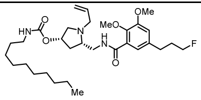	96.06
**(R, R)-cis-2d**	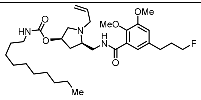	99.99

**(S, R)-trans-2i**	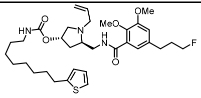	97.68
**(S, S)-cis-2i**	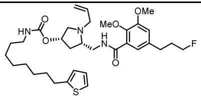	96.06
**(R, R)-cis-2i**	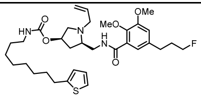	99.99

(R, S)-trans-2a GT-1-122

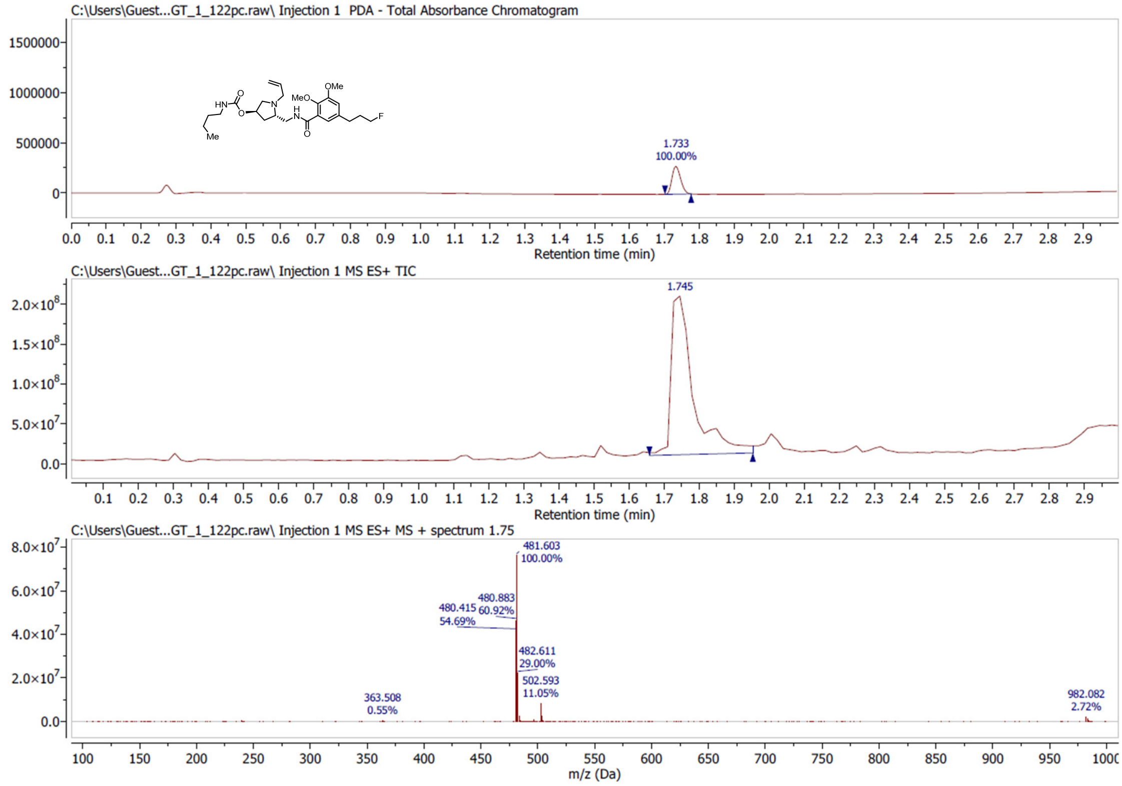

(R, S)-trans-2b GT-6-122

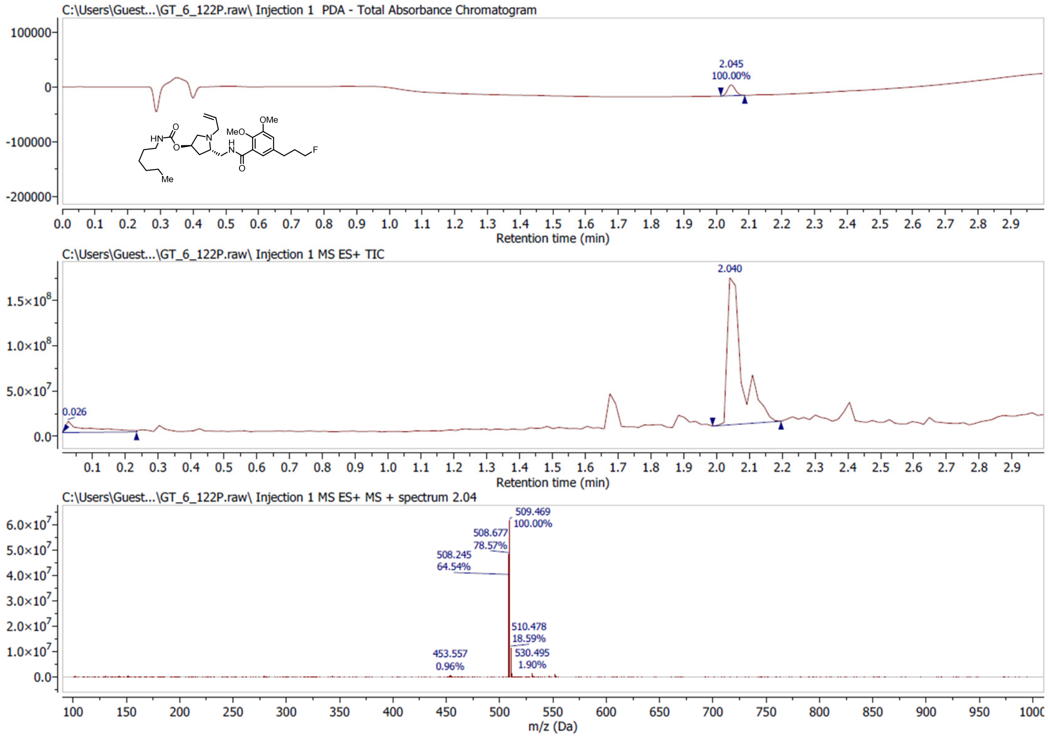

(R, S)-trans-2c GT-6-146

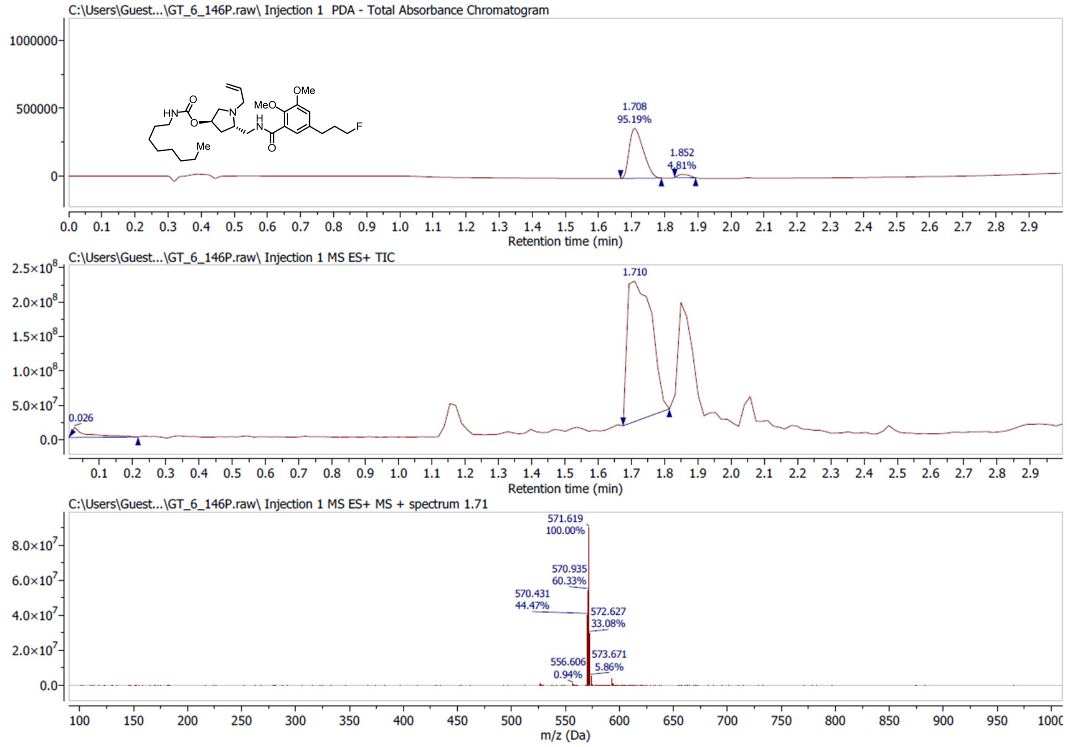

(R, S)-trans-2d GT-6-123

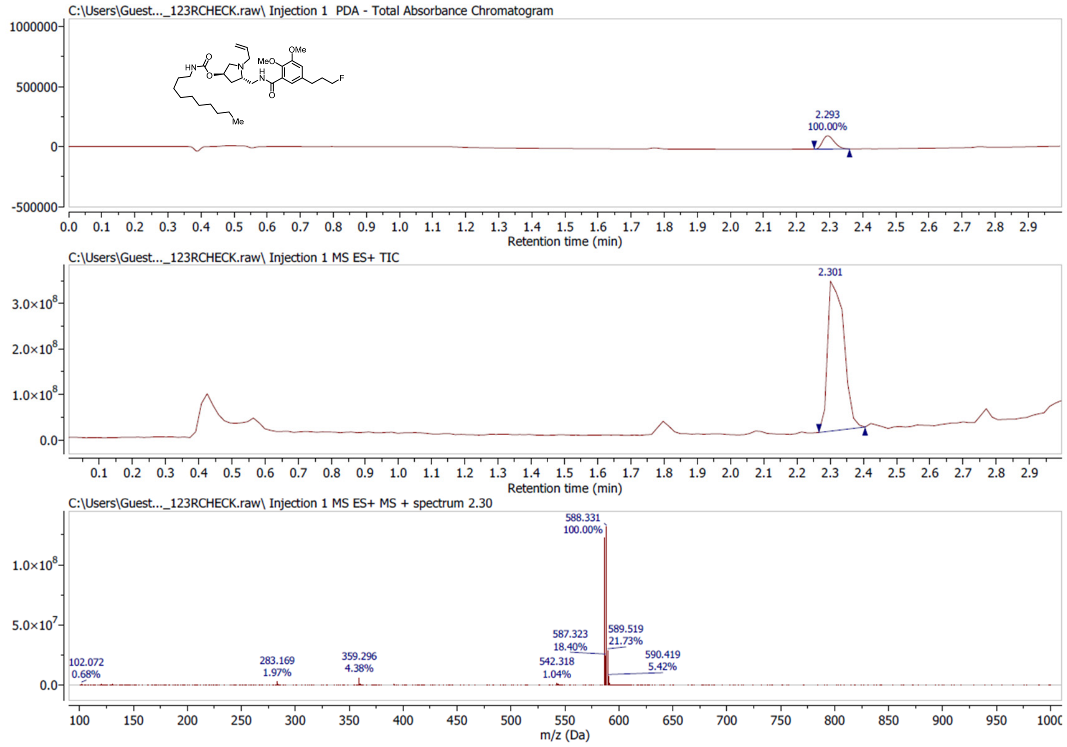

(R, S)-trans-2e GT-6-147

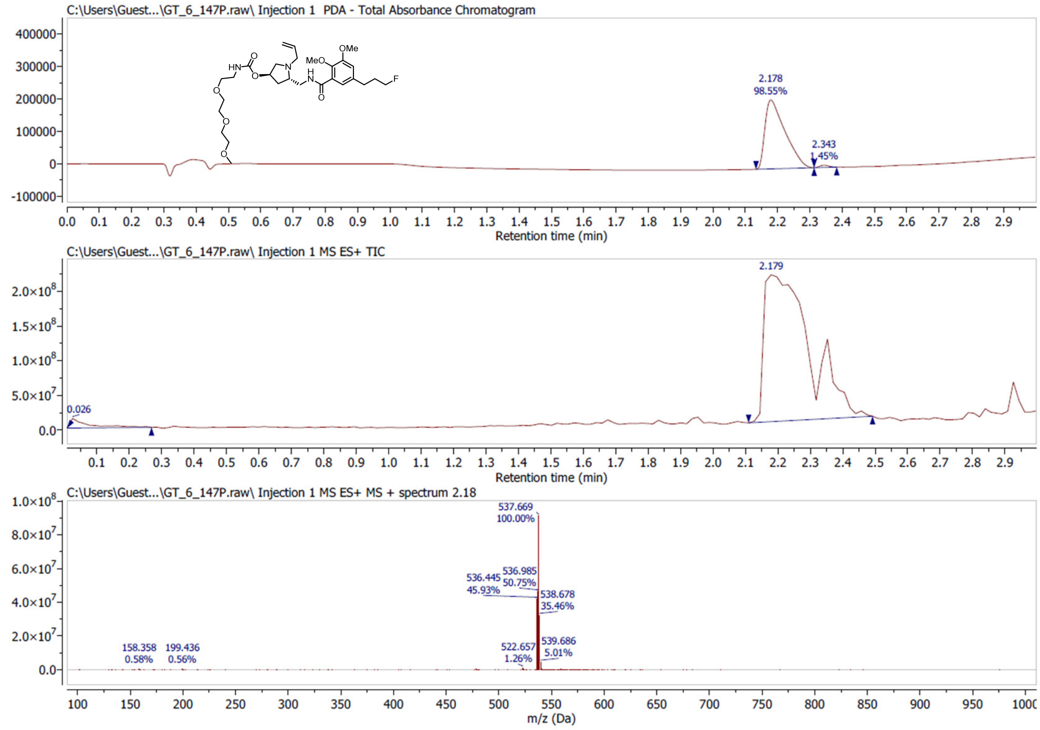

(R, S)-trans-2f GT-6-142

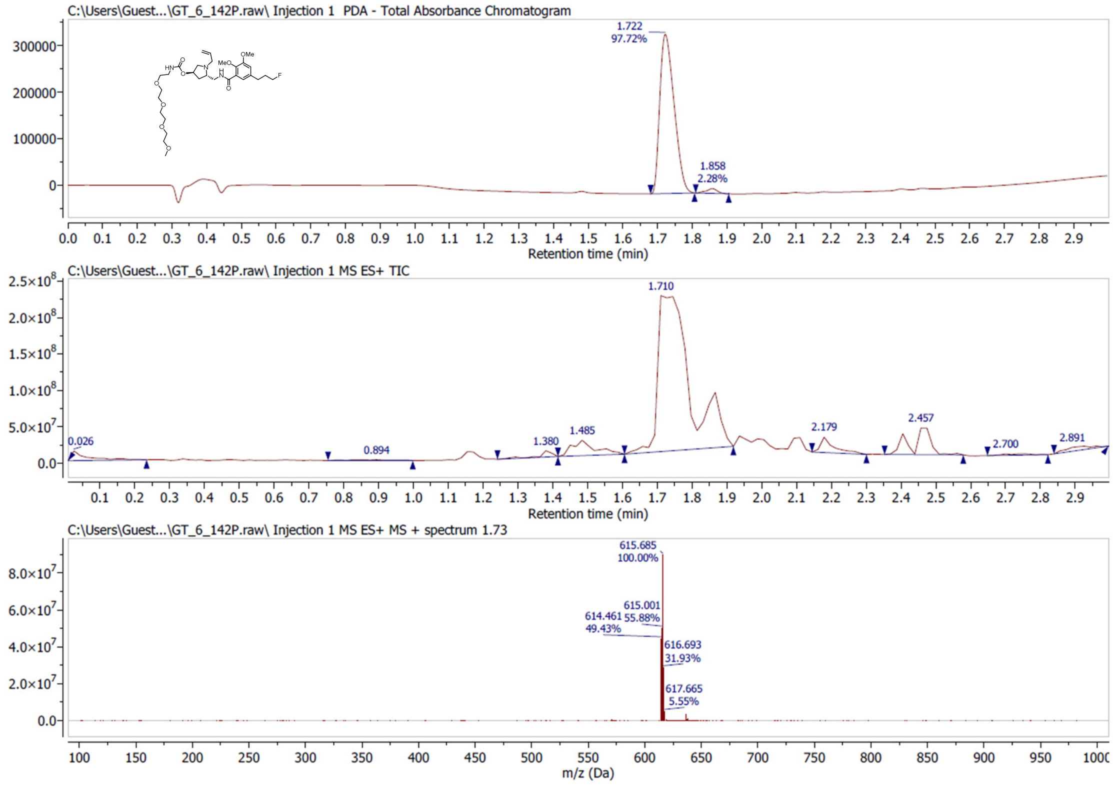

(R, S)-trans-2g GT-6-148

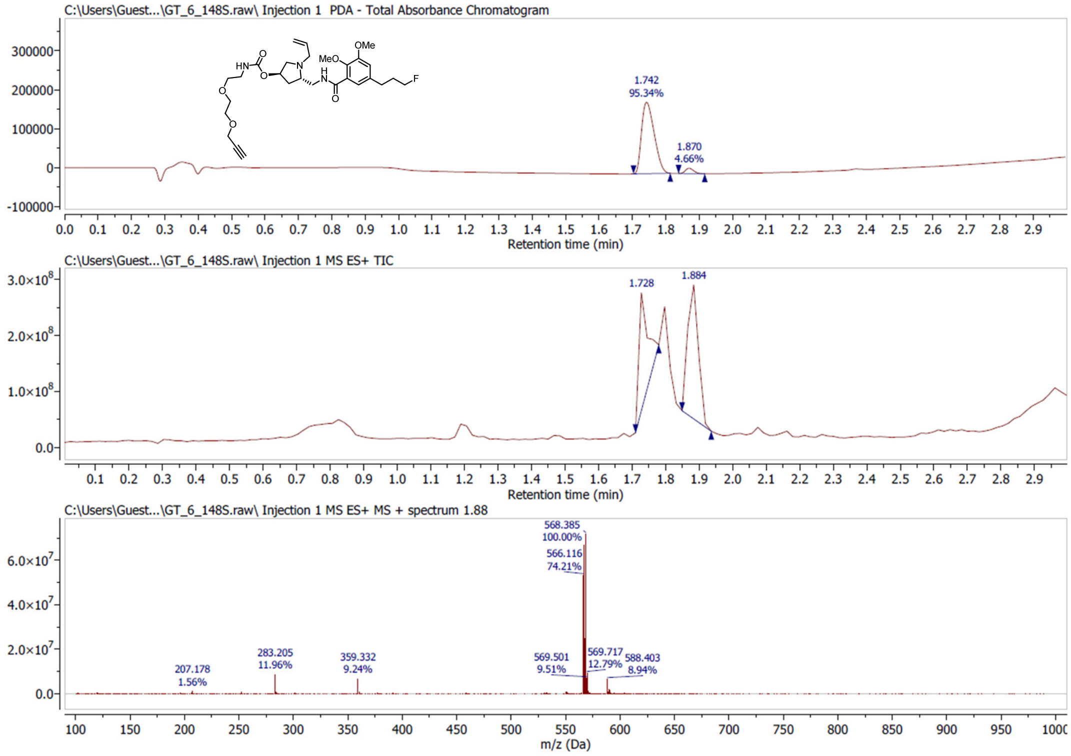

(R, S)-trans-2h GT-7-6

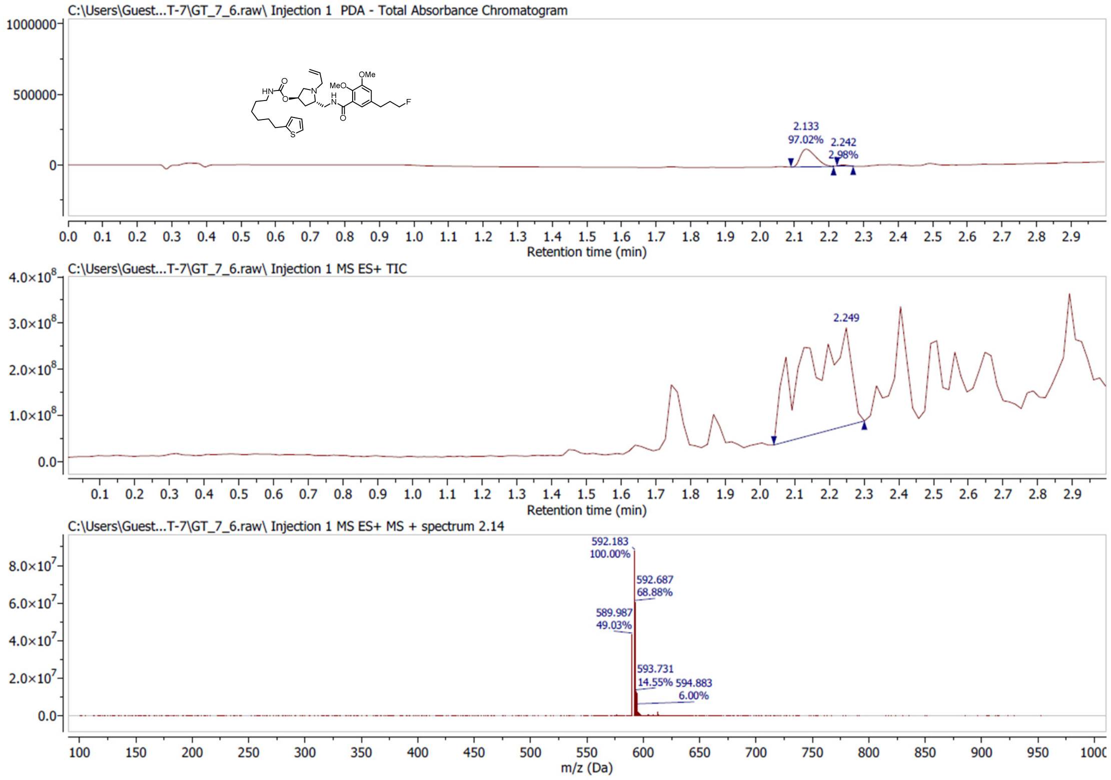

(R, S)-trans-2i GT-7-12

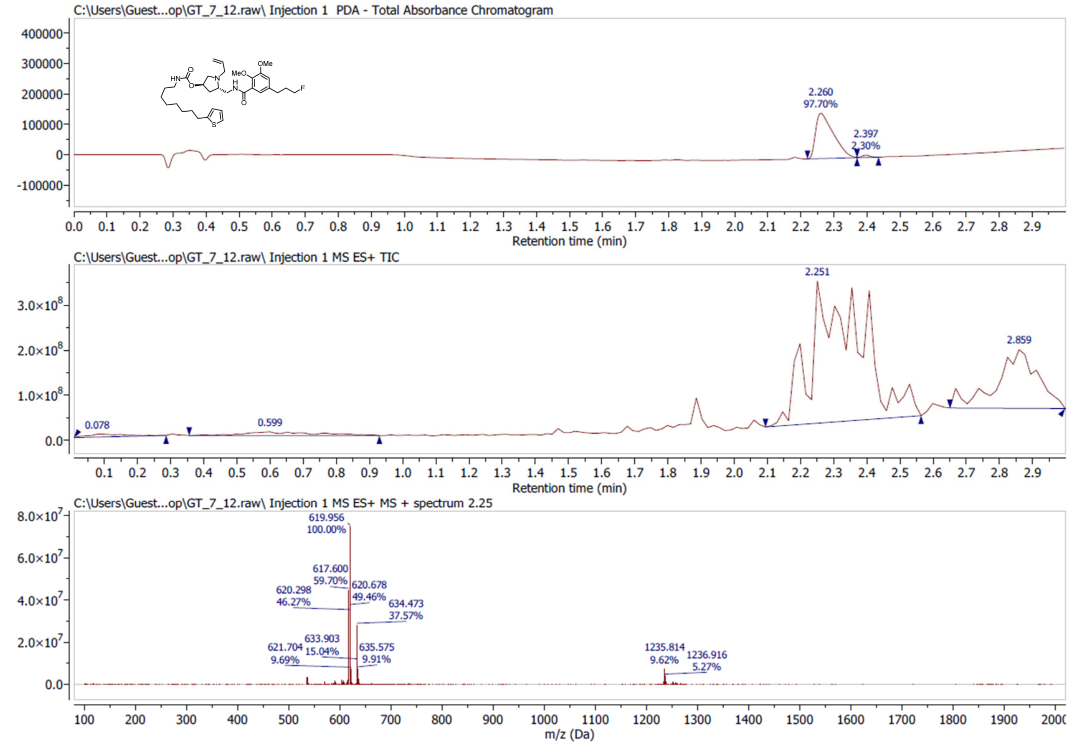

(R, S)-trans-2j GT-7-15

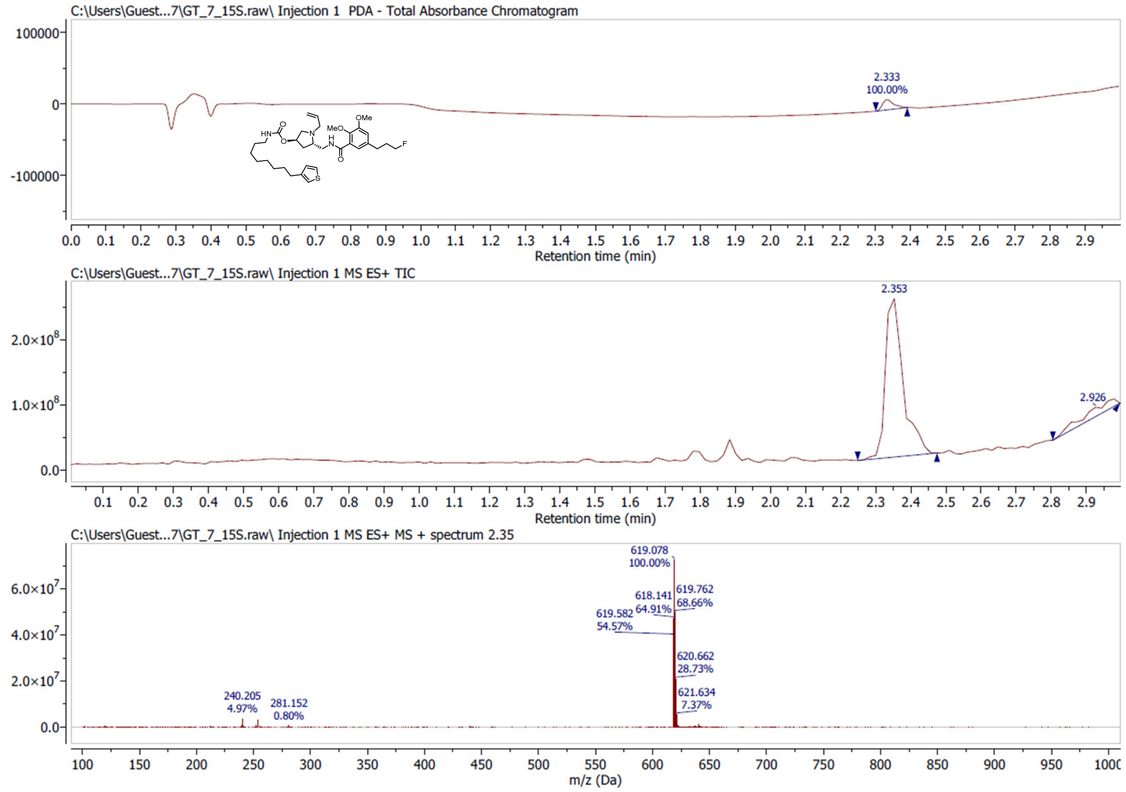

(S, R)-trans-2d GT-7-50

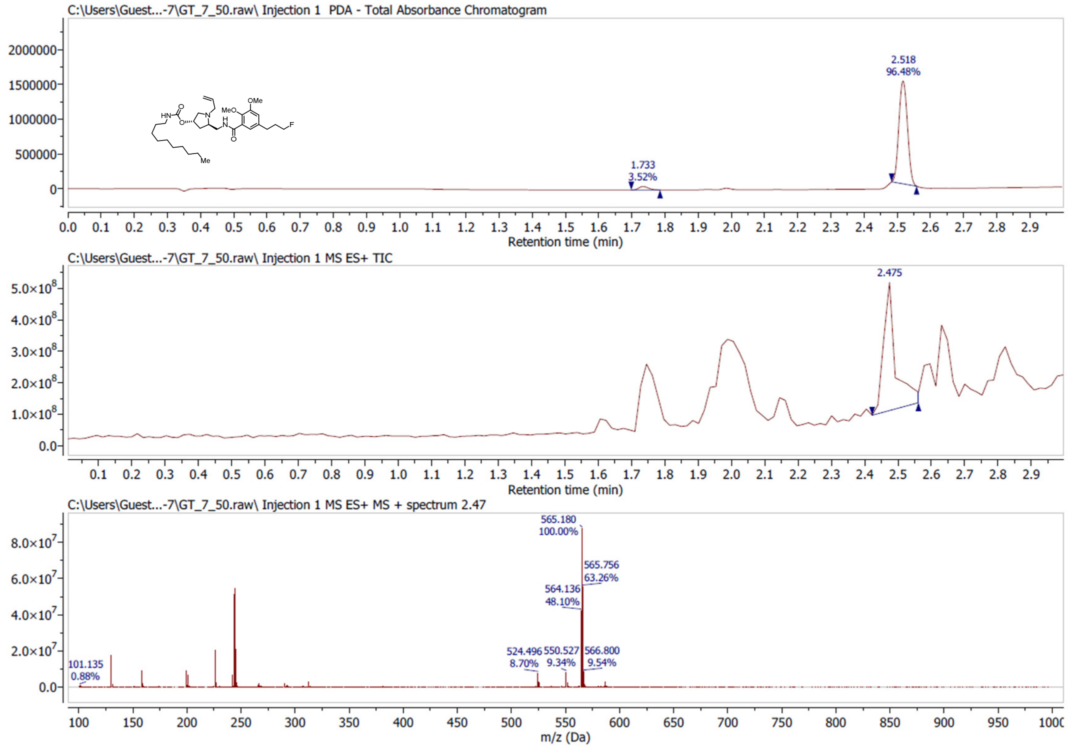

(S, S)-cis-2d GT-7-52

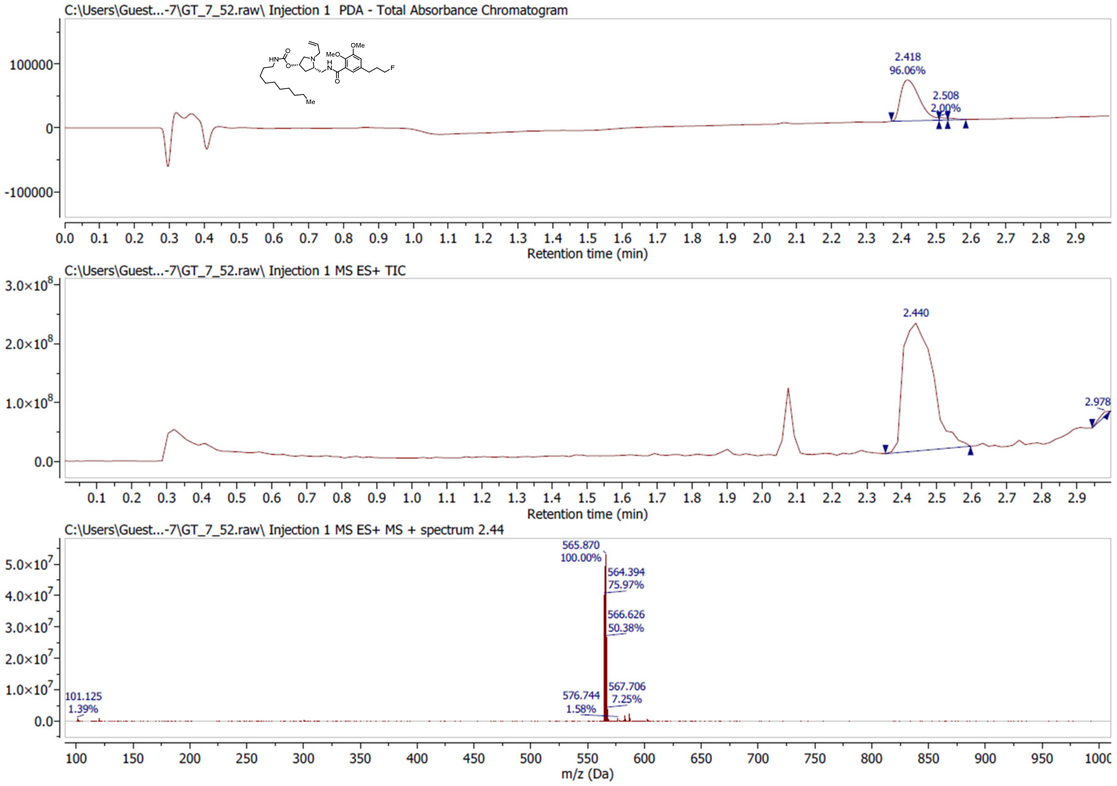

(R, R)-cis-2d GT-7-54

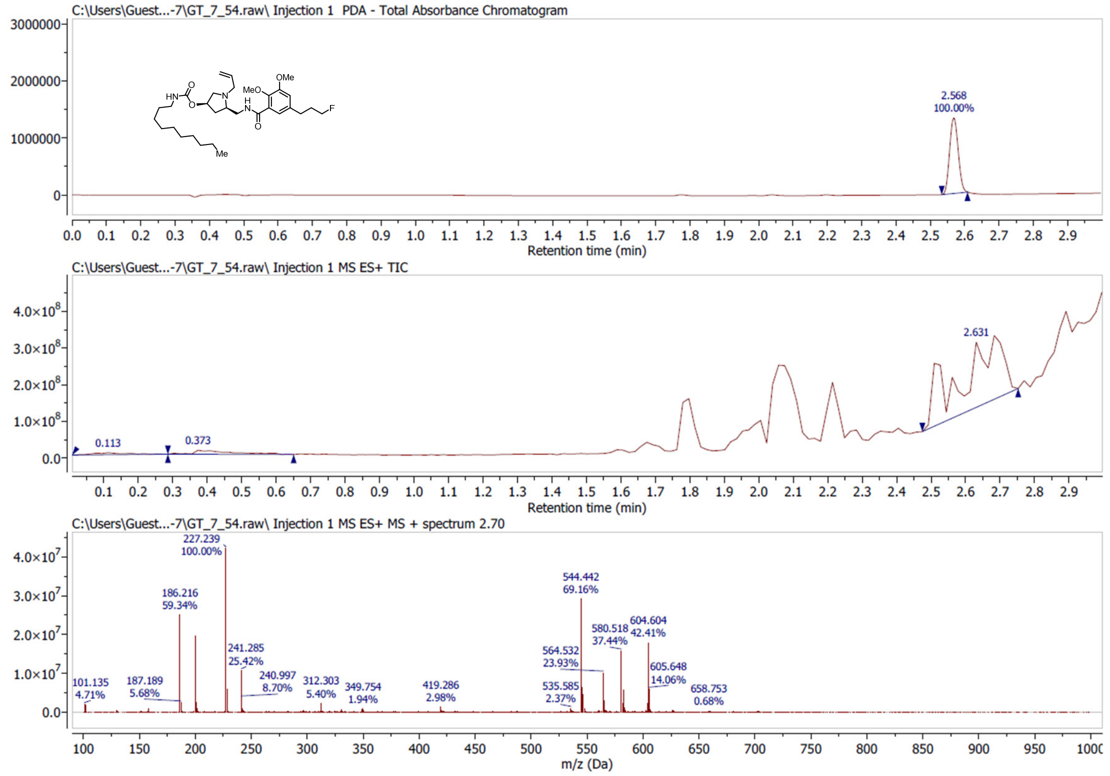

(S, R)-trans-2i GT-7-51

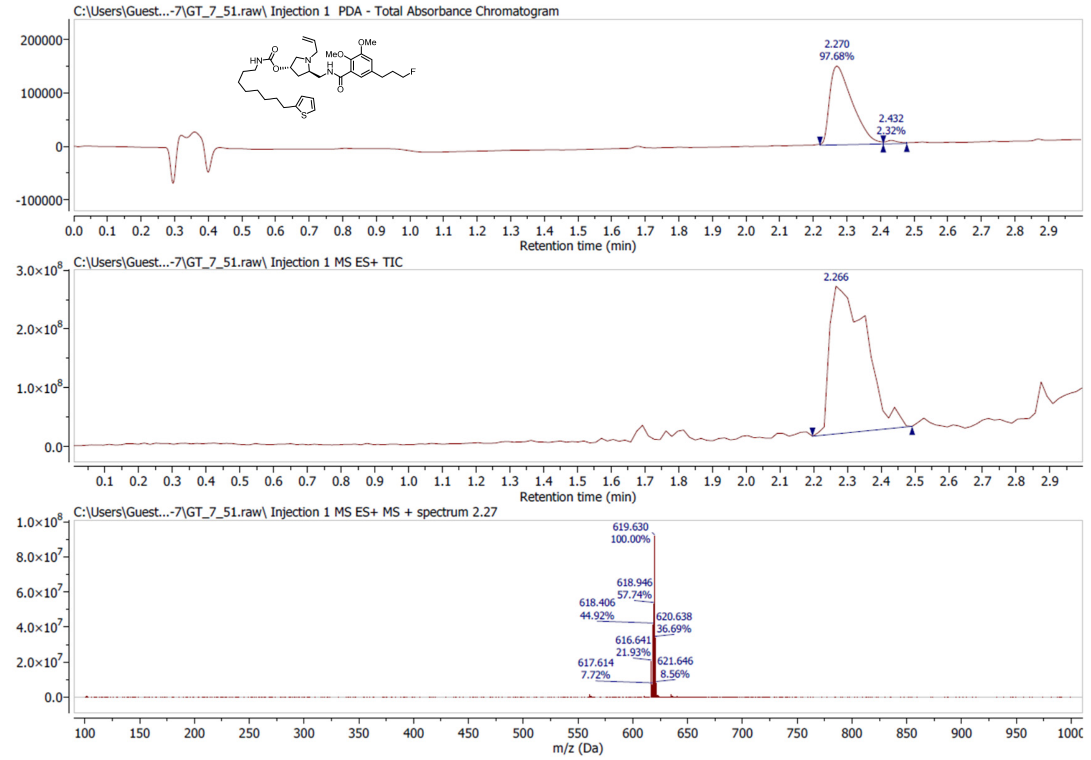

(S, S)-cis-2i GT-7-53

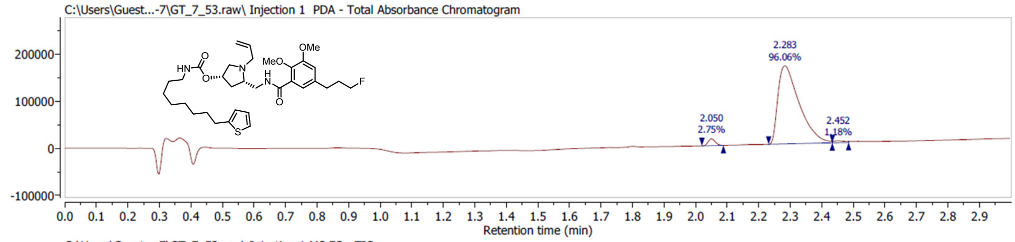

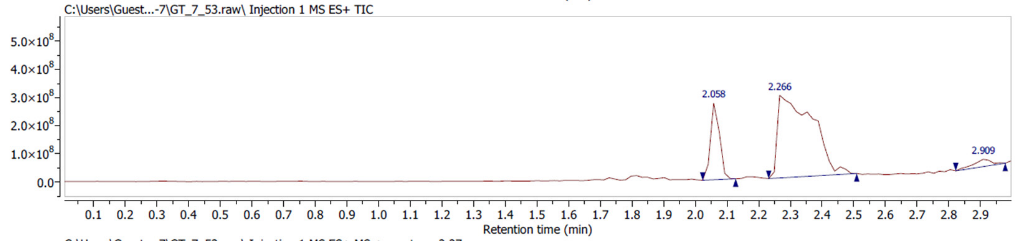

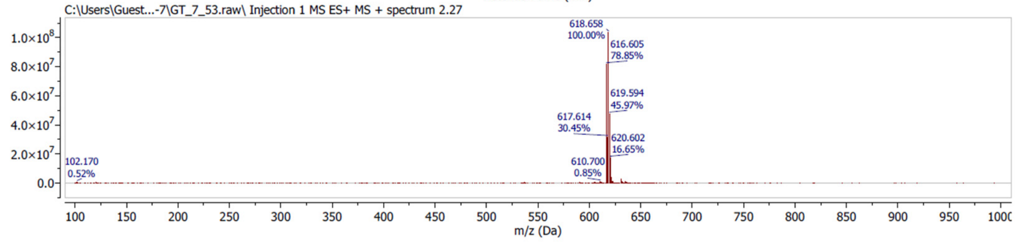

(R, R)-cis-2i GT-7-55

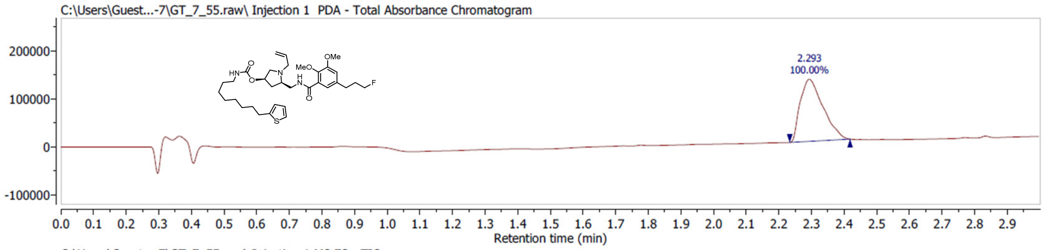

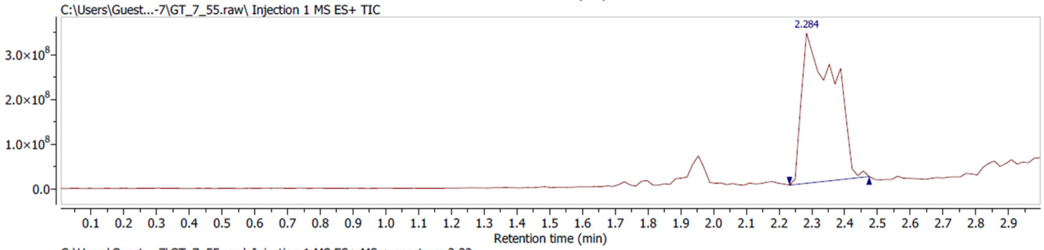

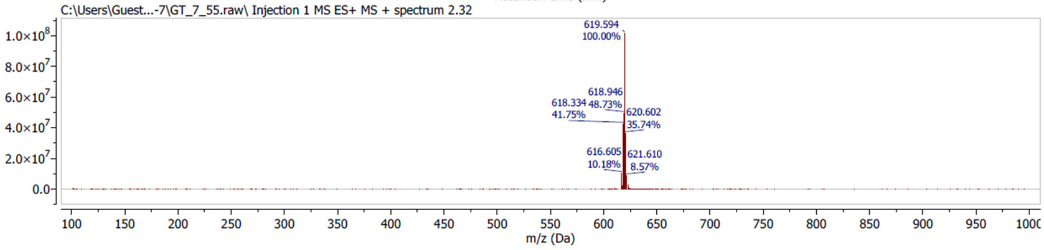

^{1}H and ^{13}C NMR Spectrum

(3R,5S)-1-allyl-5-((5-(3-fluoropropyl)-2,3-dimethoxybenzamido)methyl)pyrrolidin-3-yl butylcarbamate ***((R,S)-trans-2a)***. *GT-1-122*

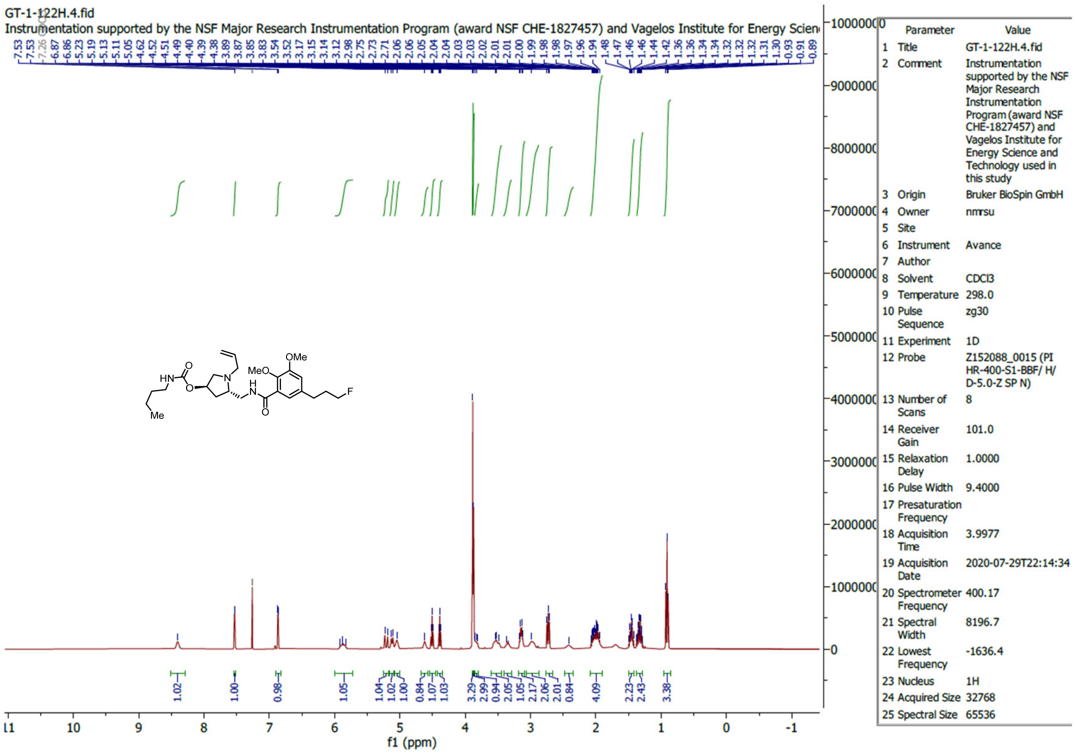

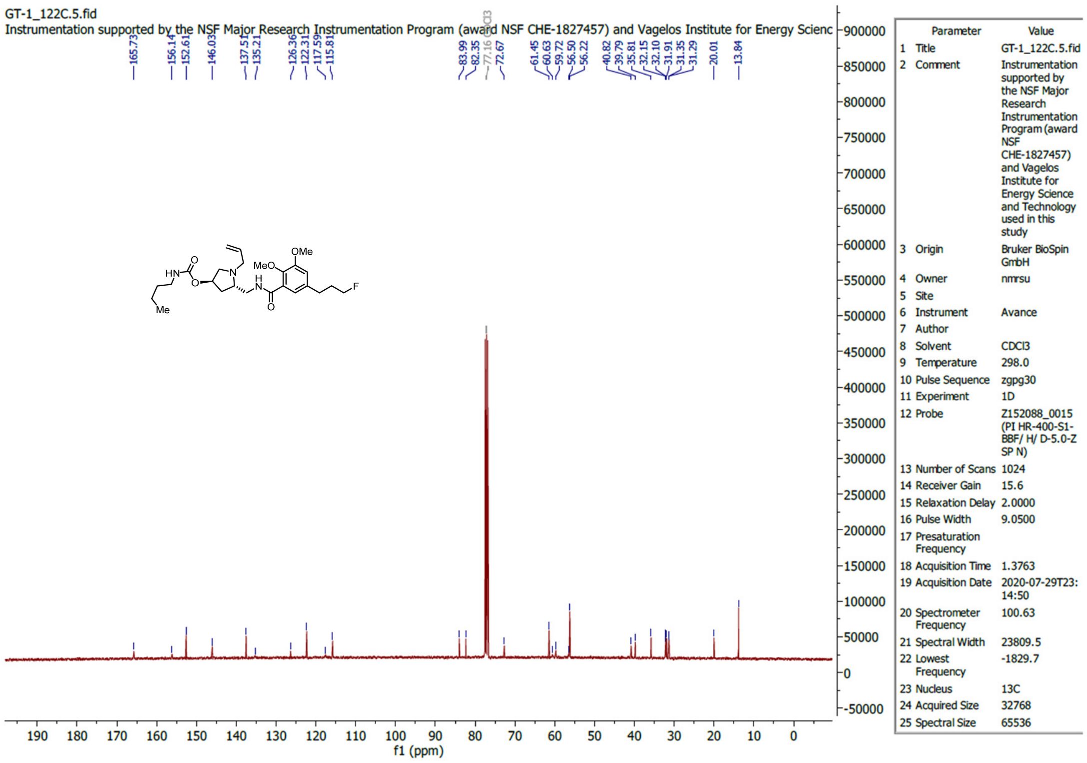

	Parameter	Value
1	Title	GT-1_122C.5.fid
2	Comment	Instrumentation supported by the NSF Major Research Instrumentation Program (award NSF CHE-1827457) and Vagelos Institute for Energy Science and Technology used in this study
3	Origin	Bruker BioSpin GmbH
4	Owner	nmrsu
5	Site	
6	Instrument	Avance
7	Author	
8	Solvent	CDCl3
9	Temperature	298.0
10	Pulse Sequence	zgpg30
11	Experiment	1D
12	Probe	Z152088_0015 (PI HR-400-S1-BBF/ H/ D-5.0-Z SP N)
13	Number of Scans	1024
14	Receiver Gain	15.6
15	Relaxation Delay	2.0000
16	Pulse Width	9.0500
17	Presaturation Frequency	
18	Acquisition Time	1.3763
19	Acquisition Date	2020-07-29T23:14:50
20	Spectrometer Frequency	100.63
21	Spectral Width	23809.5
22	Lowest Frequency	-1829.7
23	Nucleus	13C
24	Acquired Size	32768
25	Spectral Size	65536

*(3R,5S)-1-allyl-5-((5-(3-fluoropropyl)-2,3-dimethoxybenzamido)methyl)pyrrolidin-3-yl hexylcarbamate ((**R,S)-trans-2b**). GT-6-122*

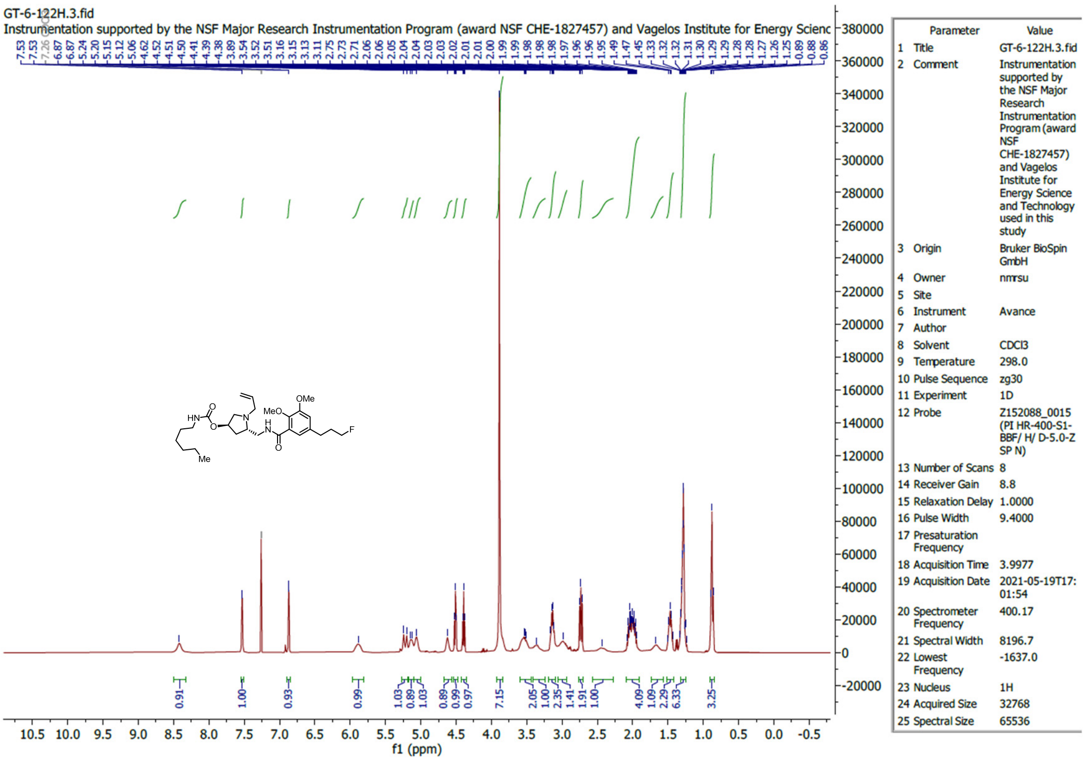

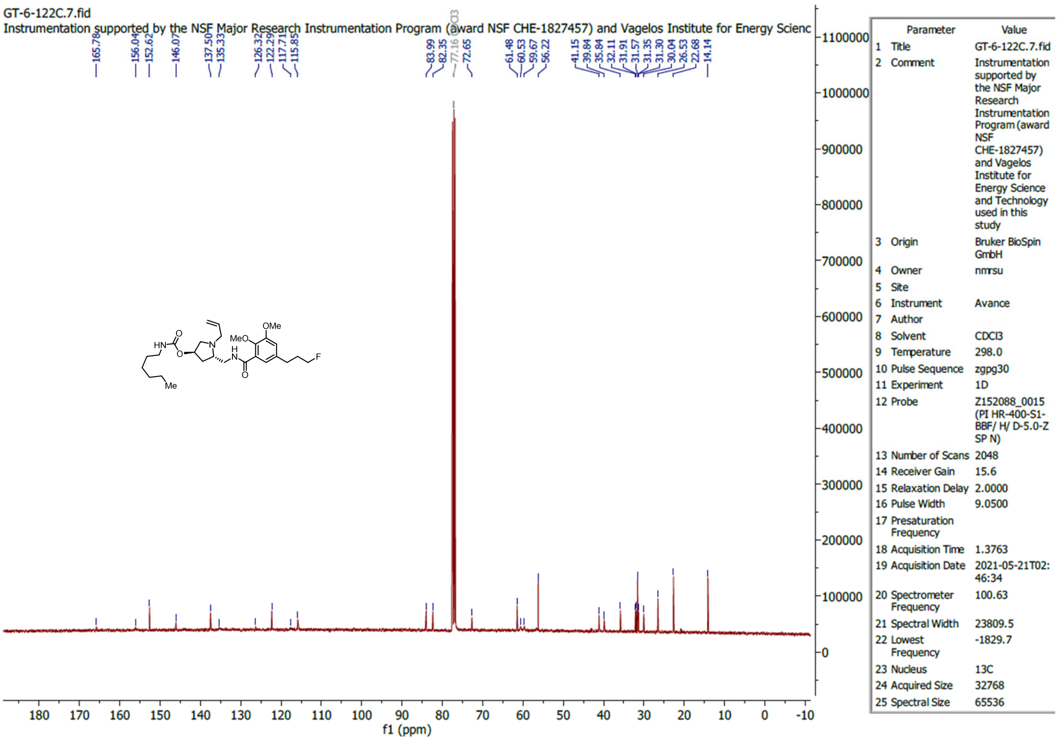

*(3R,5S)-1-allyl-5-((5-(3-fluoropropyl)-2,3-dimethoxybenzamido)methyl)pyrrolidin-3-yl octylcarbamate ((**R,S)-trans-2c**)*. GT-6-146

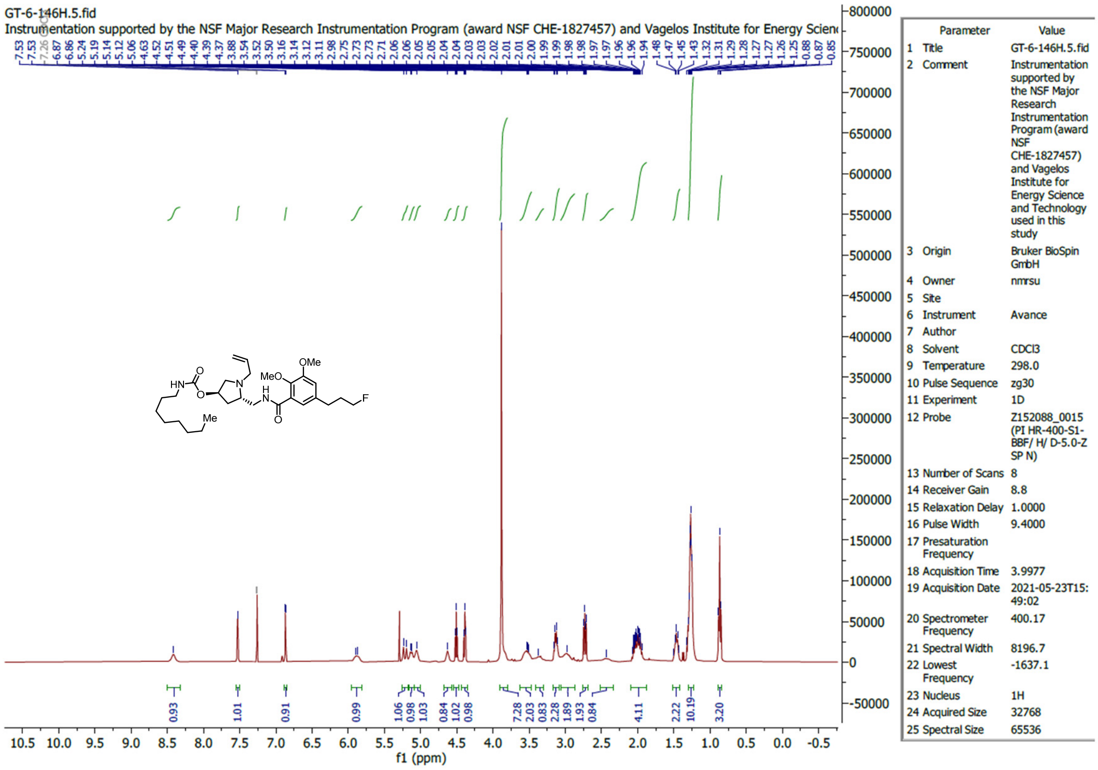

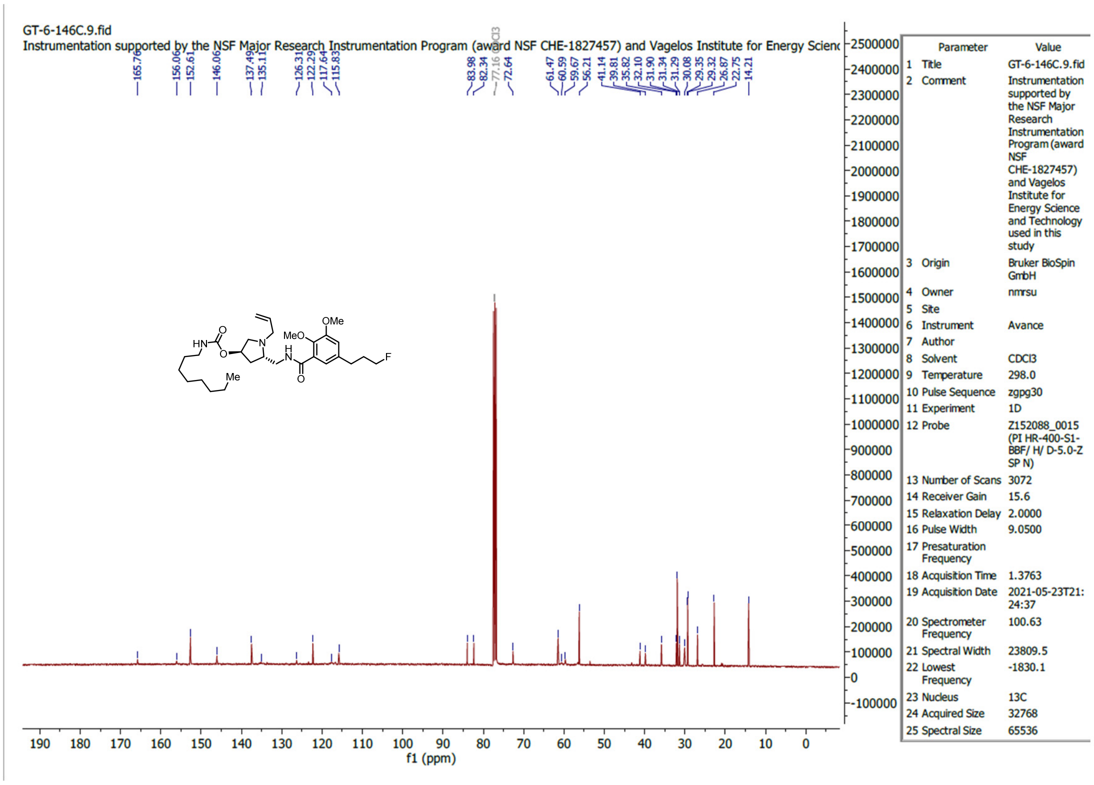

*(3R,5S)-1-allyl-5-((5-(3-fluoropropyl)-2,3-dimethoxybenzamido)methyl)pyrrolidin-3-yl decylcarbamate (**(R,S)-trans-2d**)* GT-6-123 *and (3S,5R)-1-allyl-5-((5-(3-fluoropropyl)-2,3-dimethoxybenzamido)methyl)pyrrolidin-3-yl decylcarbamate (**(S,R)-trans-2d**)*. GT-7-50

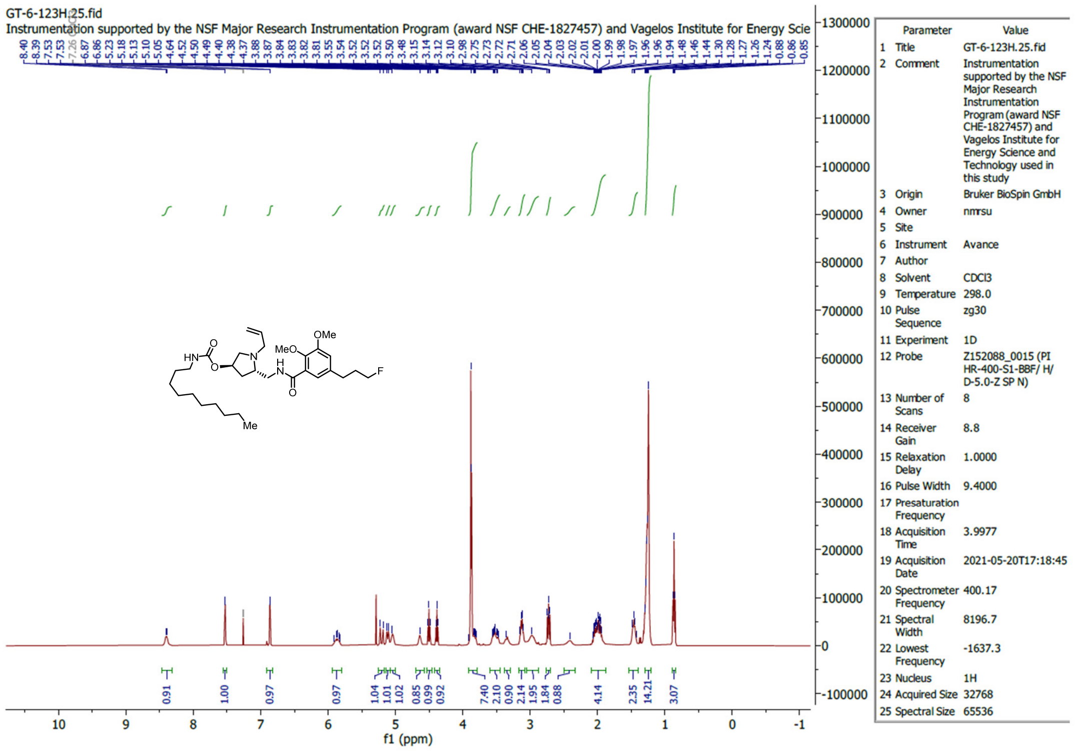

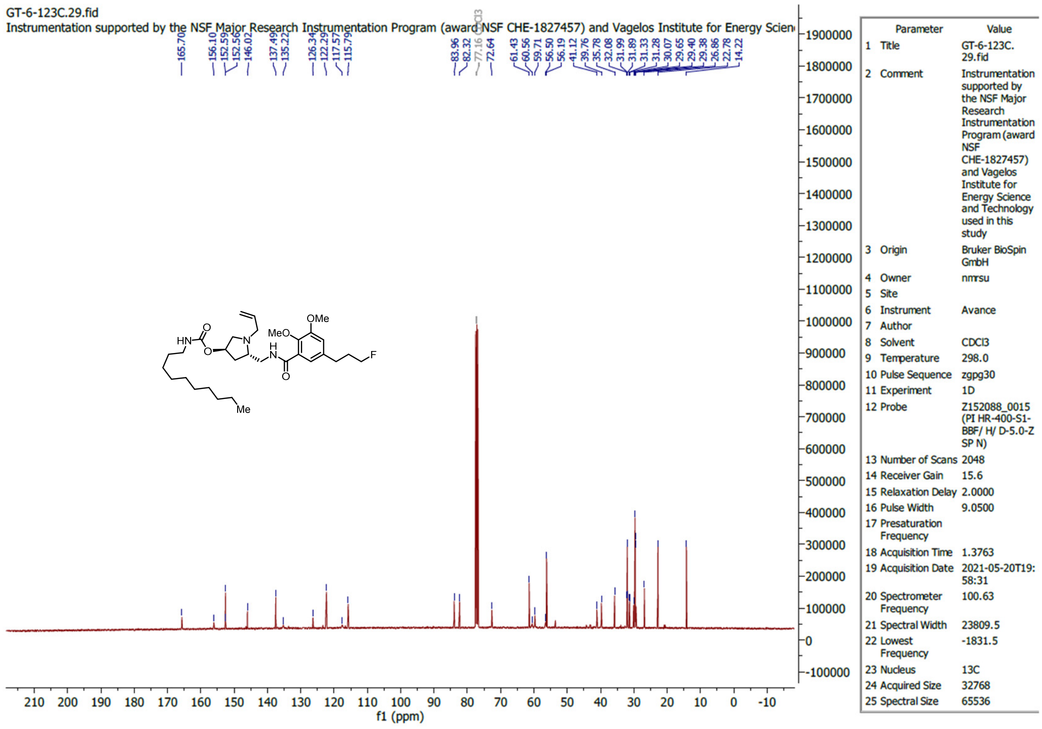

*(3R,5S)-1-allyl-5-((5-(3-fluoropropyl)-2,3-dimethoxybenzamido)methyl)pyrrolidin-3-yl (2-(2-(2-methoxyethoxy)ethoxy)ethyl)carbamate (**(R,S)-trans-2e**)*. GT-6-147

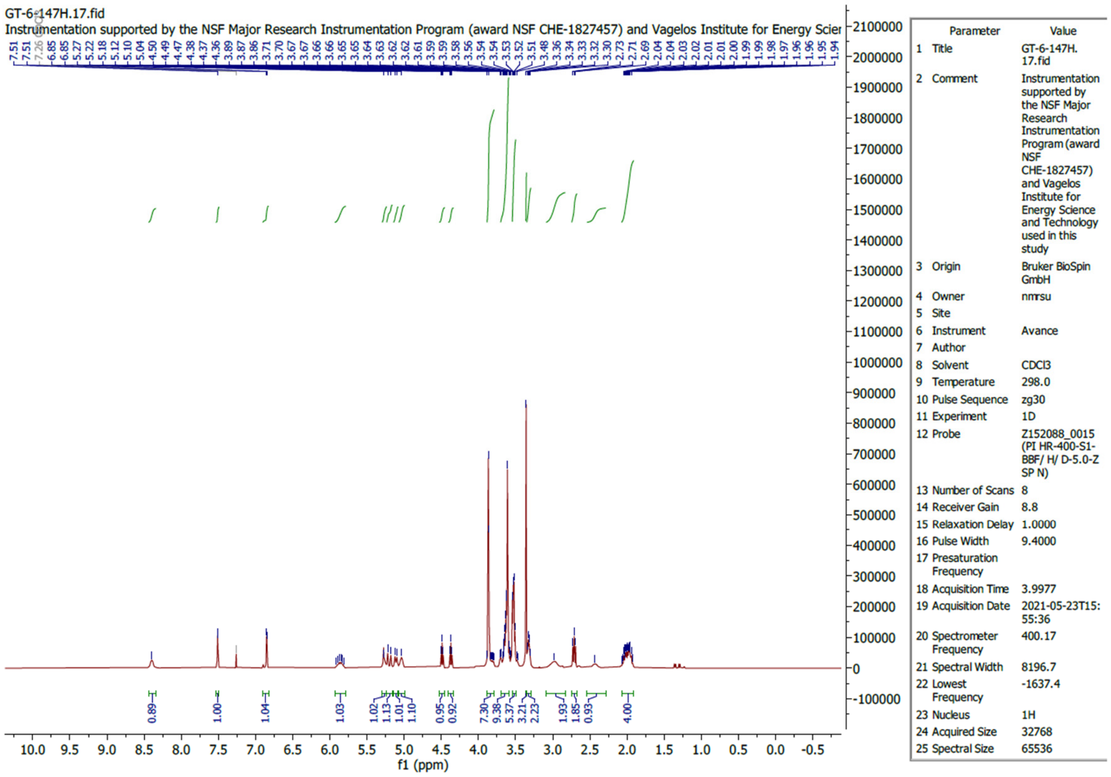

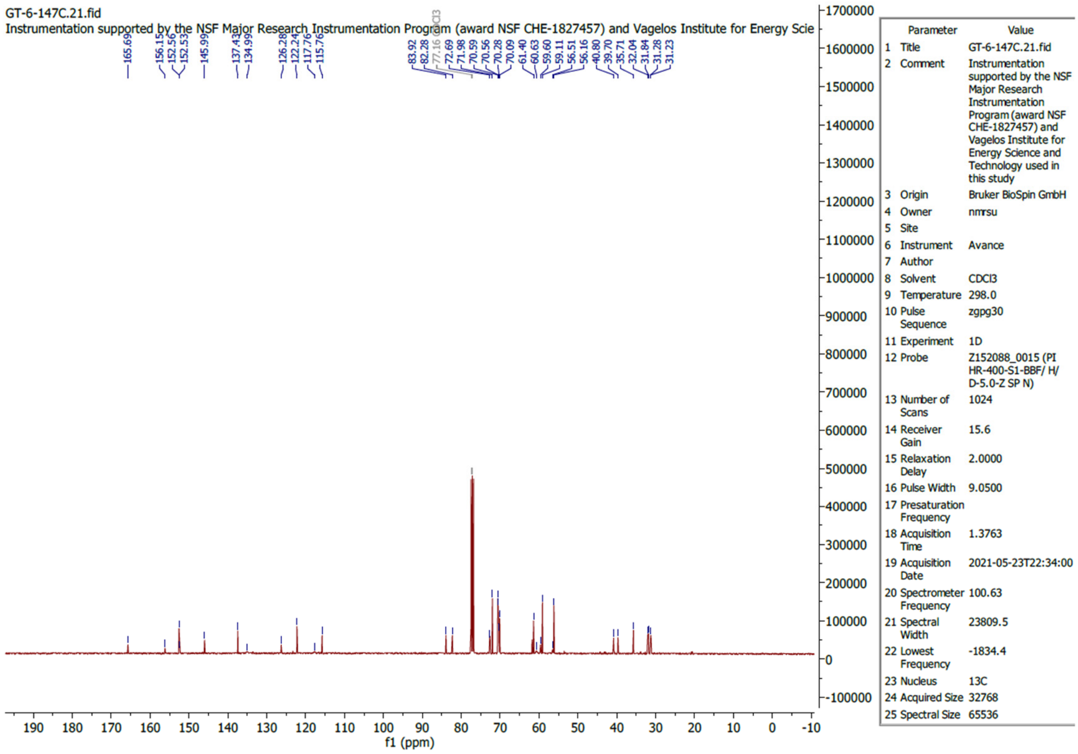

(3R,5S)-1-allyl-5-((5-(3-fluoropropyl)-2,3-dimethoxybenzamido)methyl)pyrrolidin-3-yl (2,5,8,11-tetraoxatridecan-13-yl)carbamate (***(R,S)-trans-2f***). GT-6-142

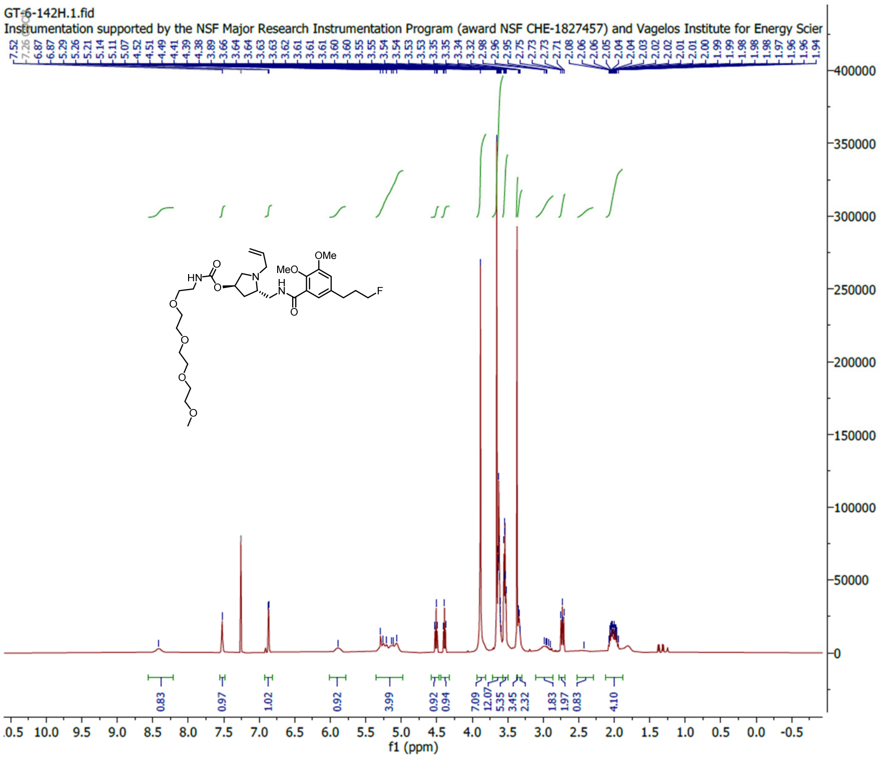

	Parameter	Value
1	Title	GT-6-142H.1.fid
2	Comment	Instrumentation supported by the NSF Major Research Instrumentation Program (award NSF CHE-1827457) and Vagelos Institute for Energy Science and Technology used in this study
3	Origin	Bruker BioSpin GmbH
4	Owner	nmrsu
5	Site	
6	Instrument	Avance
7	Author	
8	Solvent	CDCl3
9	Temperature	298.0
10	Pulse Sequence	zg30
11	Experiment	1D
12	Probe	Z152088_0015 (PI HR-400-S1-BBF/ H/ D-5.0-Z SP N)
13	Number of Scans	8
14	Receiver Gain	8.8
15	Relaxation Delay	1.0000
16	Pulse Width	9.4000
17	Presaturation Frequency	
18	Acquisition Time	3.9977
19	Acquisition Date	2021-05-30T17:23:26
20	Spectrometer Frequency	400.17
21	Spectral Width	8196.7
22	Lowest Frequency	-1637.1
23	Nucleus	1H
24	Acquired Size	32768
25	Spectral Size	65536

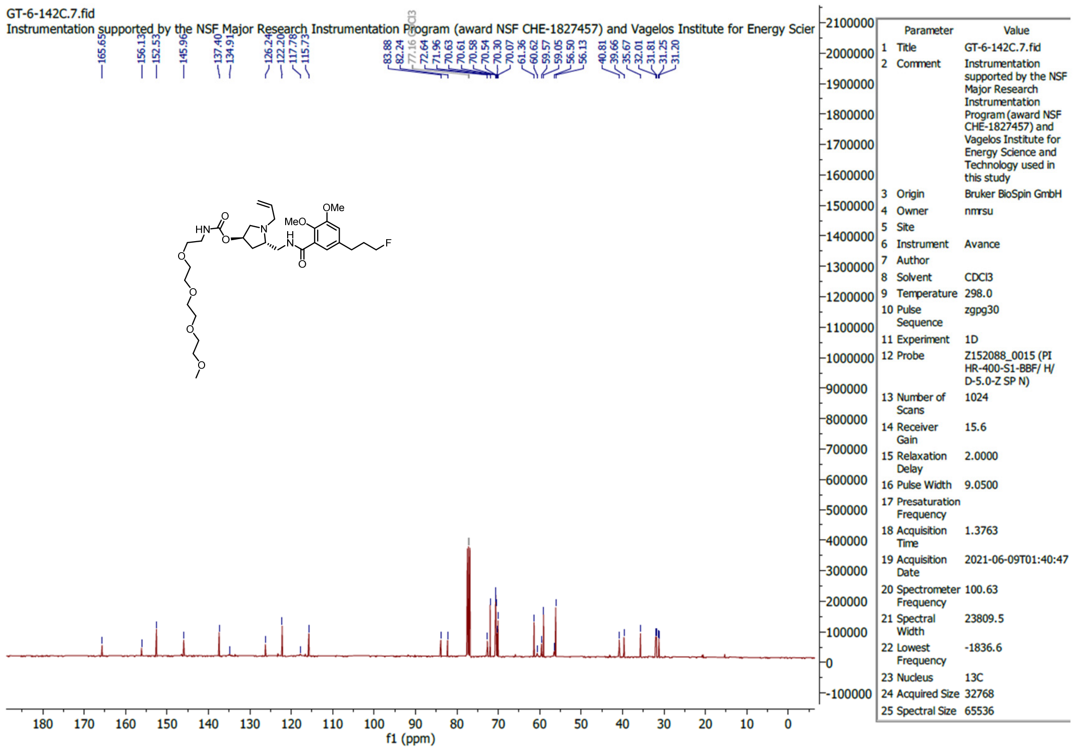

	Parameter	Value
1	Title	GT-6-142C.7.fid
2	Comment	Instrumentation supported by the NSF Major Research Instrumentation Program (award NSF CHE-1827457) and Vagelos Institute for Energy Science and Technology used in this study
3	Origin	Bruker BioSpin GmbH
4	Owner	nmrsu
5	Site	
6	Instrument	Avance
7	Author	
8	Solvent	CDCl3
9	Temperature	298.0
10	Pulse Sequence	zgpg30
11	Experiment	1D
12	Probe	Z152088_0015 (PI HR-400-S1-BBF/ H/ D-5.0-Z SP N)
13	Number of Scans	1024
14	Receiver Gain	15.6
15	Relaxation Delay	2.0000
16	Pulse Width	9.0500
17	Presaturation Frequency	
18	Acquisition Time	1.3763
19	Acquisition Date	2021-06-09T01:40:47
20	Spectrometer Frequency	100.63
21	Spectral Width	23809.5
22	Lowest Frequency	-1836.6
23	Nucleus	13C
24	Acquired Size	32768
25	Spectral Size	65536

*(3R,5S)-1-allyl-5-((5-(3-fluoropropyl)-2,3-dimethoxybenzamido)methyl)pyrrolidin-3-yl (2-(2-(prop-2-yn-1-yloxy)ethoxy)ethyl)carbamate (**(R,S)-trans-2g**)*. GT-6-148

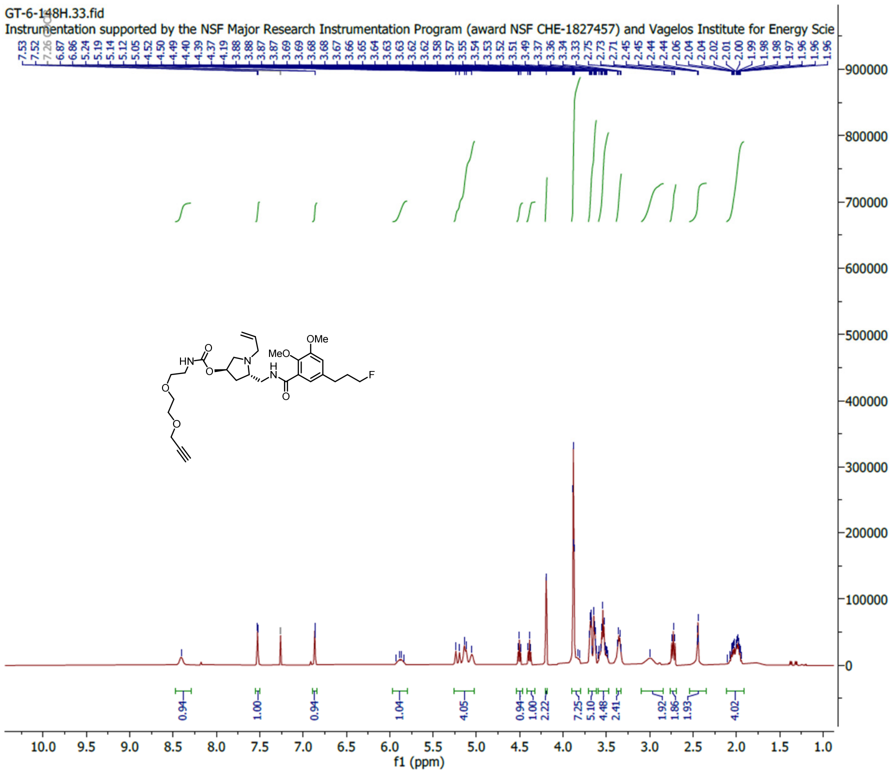

	Parameter	Value
1	Title	GT-6-148H.33.fid
2	Comment	Instrumentation supported by the NSF Major Research Instrumentation Program (award NSF CHE-1827457) and Vagelos Institute for Energy Science and Technology used in this study
3	Origin	Bruker BioSpin GmbH
4	Owner	nmrsu
5	Site	
6	Instrument	Avance
7	Author	
8	Solvent	CDCl3
9	Temperature	298.0
10	Pulse Sequence	zg30
11	Experiment	1D
12	Probe	Z152088_0015 (PI HR-400-S1-BBF/ H/ D-5.0-Z SP N)
13	Number of Scans	8
14	Receiver Gain	8.8
15	Relaxation Delay	1.0000
16	Pulse Width	9.4000
17	Presaturation Frequency	
18	Acquisition Time	3.9977
19	Acquisition Date	2021-05-25T16:55:47
20	Spectrometer Frequency	400.17
21	Spectral Width	8196.7
22	Lowest Frequency	-1637.0
23	Nucleus	1H
24	Acquired Size	32768
25	Spectral Size	65536

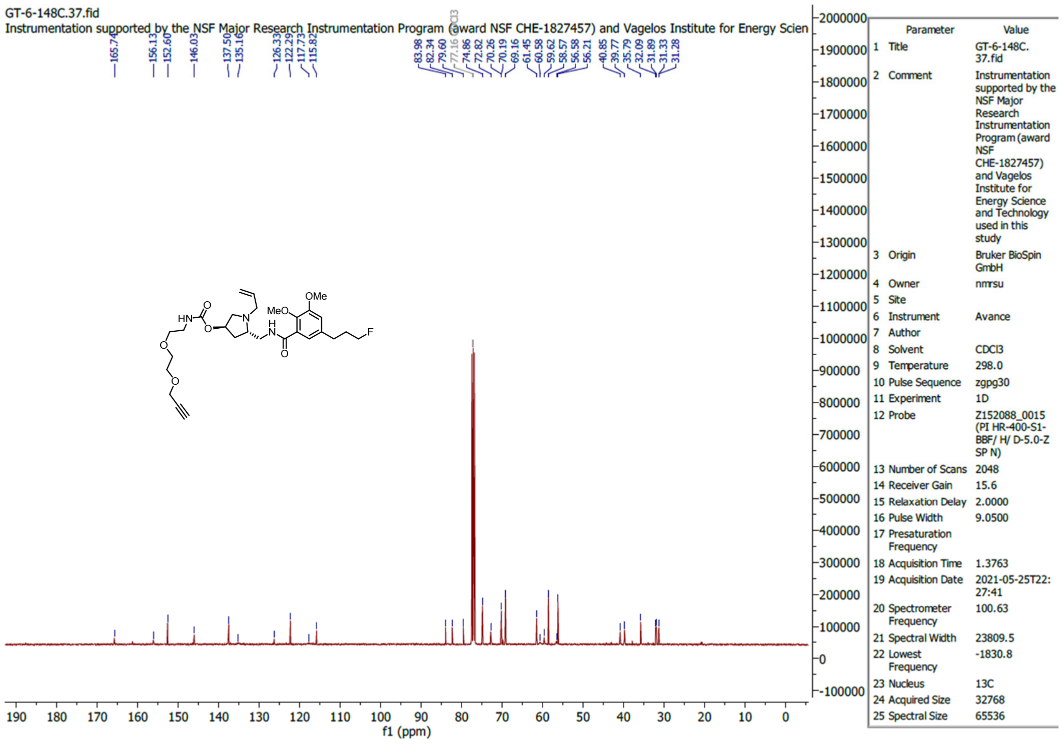

	Parameter	Value
1	Title	GT-6-148C.37.fid
2	Comment	Instrumentation supported by the NSF Major Research Instrumentation Program (award NSF CHE-1827457) and Vagelos Institute for Energy Science and Technology used in this study
3	Origin	Bruker BioSpin GmbH
4	Owner	nmrsu
5	Site	
6	Instrument	Avance
7	Author	
8	Solvent	CDCl3
9	Temperature	298.0
10	Pulse Sequence	zgpg30
11	Experiment	1D
12	Probe	Z152088_0015 (PI HR-400-S1-BBF/ H/ D-5.0-Z SP N)
13	Number of Scans	2048
14	Receiver Gain	15.6
15	Relaxation Delay	2.0000
16	Pulse Width	9.0500
17	Presaturation Frequency	
18	Acquisition Time	1.3763
19	Acquisition Date	2021-05-25T22:27:41
20	Spectrometer Frequency	100.63
21	Spectral Width	23809.5
22	Lowest Frequency	-1830.8
23	Nucleus	13C
24	Acquired Size	32768
25	Spectral Size	65536

(3R,5S)-1-allyl-5-((5-(3-fluoropropyl)-2,3-dimethoxybenzamido)methyl)pyrrolidin-3-yl (6-(thiophen-2-yl)hexyl)carbamate (***(R,S)-trans-2h***) GT-7-6

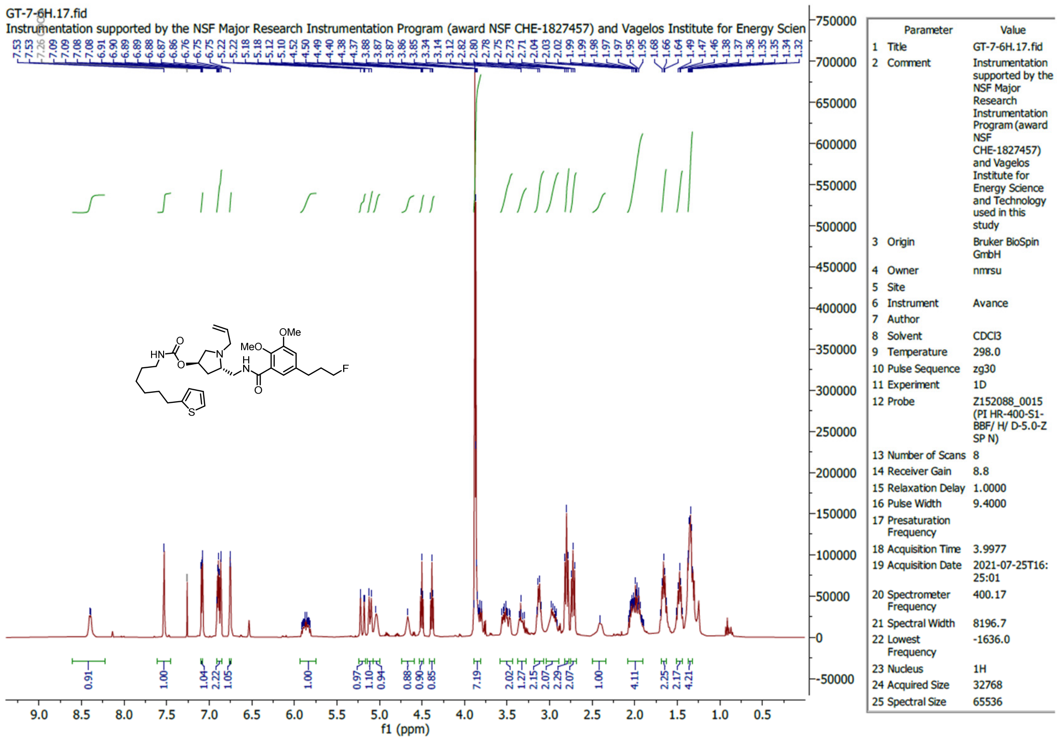

	Parameter	Value
1	Title	GT-7-6H.17.fid
2	Comment	Instrumentation supported by the NSF Major Research Instrumentation Program (award NSF CHE-1827457) and Vagelos Institute for Energy Science and Technology used in this study
3	Origin	Bruker BioSpin GmbH
4	Owner	nmrsu
5	Site	
6	Instrument	Avance
7	Author	
8	Solvent	CDCl3
9	Temperature	298.0
10	Pulse Sequence	zg30
11	Experiment	1D
12	Probe	Z152088_0015 (PI HR-400-S1-BBF/ H/ D-5.0-Z SP N)
13	Number of Scans	8
14	Receiver Gain	8.8
15	Relaxation Delay	1.0000
16	Pulse Width	9.4000
17	Presaturation Frequency	
18	Acquisition Time	3.9977
19	Acquisition Date	2021-07-25T16:25:01
20	Spectrometer Frequency	400.17
21	Spectral Width	8196.7
22	Lowest Frequency	-1636.0
23	Nucleus	1H
24	Acquired Size	32768
25	Spectral Size	65536

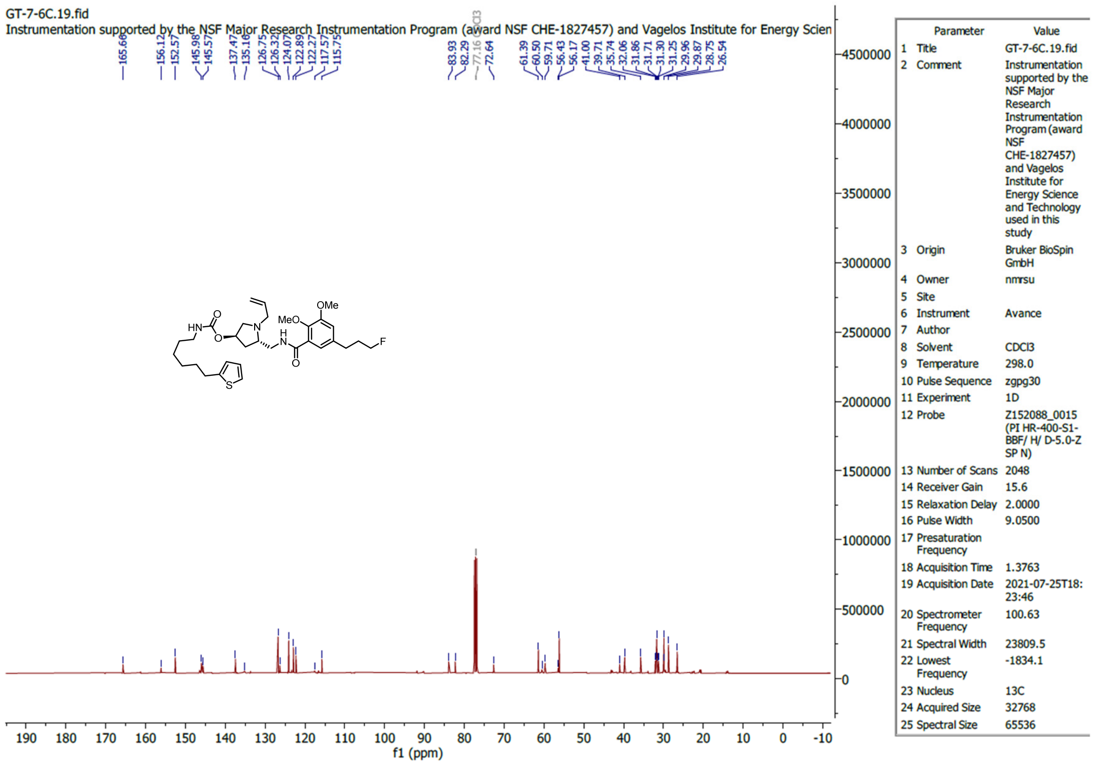

	Parameter	Value
1	Title	GT-7-6C.19.fid
2	Comment	Instrumentation supported by the NSF Major Research Instrumentation Program (award NSF CHE-1827457) and Vagelos Institute for Energy Science and Technology used in this study
3	Origin	Bruker BioSpin GmbH
4	Owner	nmrsu
5	Site	
6	Instrument	Avance
7	Author	
8	Solvent	CDCl3
9	Temperature	298.0
10	Pulse Sequence	zgpg30
11	Experiment	1D
12	Probe	Z152088_0015 (PI HR-400-S1-BBF/ H/ D-5.0-Z SP N)
13	Number of Scans	2048
14	Receiver Gain	15.6
15	Relaxation Delay	2.0000
16	Pulse Width	9.0500
17	Presaturation Frequency	
18	Acquisition Time	1.3763
19	Acquisition Date	2021-07-25T18:23:46
20	Spectrometer Frequency	100.63
21	Spectral Width	23809.5
22	Lowest Frequency	-1834.1
23	Nucleus	13C
24	Acquired Size	32768
25	Spectral Size	65536

*(3R,5S)-1-allyl-5-((5-(3-fluoropropyl)-2,3-dimethoxybenzamido)methyl)pyrrolidin-3-yl (8-(thiophen-2-yl)octyl)carbamate (**(R,S)-trans-2i**)* GT-7-12 *or (3S,5R)-1-allyl-5-((5-(3-fluoropropyl)-2,3-dimethoxybenzamido)methyl)pyrrolidin-3-yl (8-(thiophen-2-yl)octyl)carbamate (**(S,R)-trans-2i**)*. GT-7-51

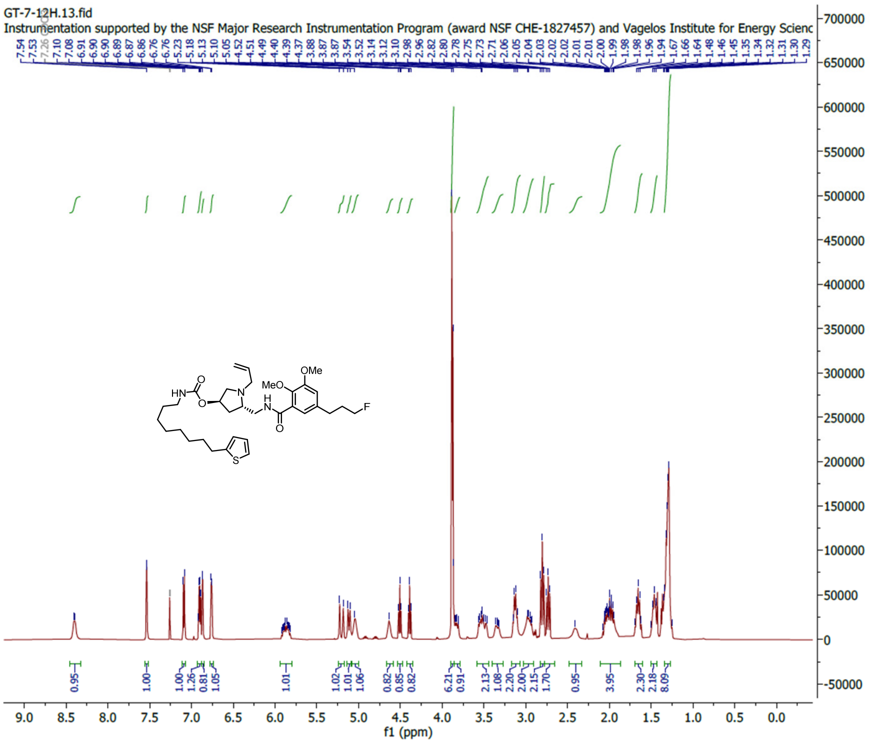

Parameter		Value
1	Title	GT-7-12H.13.fid
2	Comment	Instrumentation supported by the NSF Major Research Instrumentation Program (award NSF CHE-1827457) and Vagelos Institute for Energy Science and Technology used in this study
3	Origin	Bruker BioSpin GmbH
4	Owner	nmrsu
5	Site	
6	Instrument	Avance
7	Author	
8	Solvent	CDCl3
9	Temperature	298.0
10	Pulse Sequence	zg30
11	Experiment	1D
12	Probe	Z152088_0015 (PI HR-400-S1-BBF/ H/ D-5.0-Z SP N)
13	Number of Scans	8
14	Receiver Gain	8.8
15	Relaxation Delay	1.0000
16	Pulse Width	9.4000
17	Presaturation Frequency	
18	Acquisition Time	3.9977
19	Acquisition Date	2021-08-10T16:37:25
20	Spectrometer Frequency	400.17
21	Spectral Width	8196.7
22	Lowest Frequency	-1637.0
23	Nucleus	1H
24	Acquired Size	32768
25	Spectral Size	65536

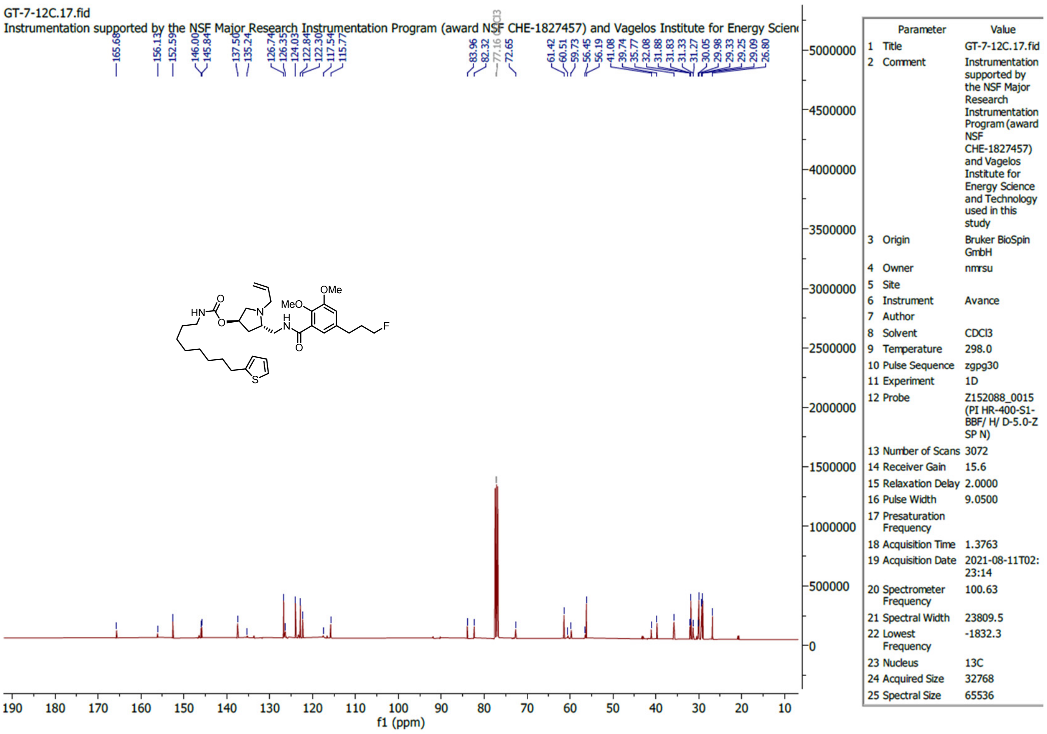

Parameter		Value
1	Title	GT-7-12C.17.fid
2	Comment	Instrumentation supported by the NSF Major Research Instrumentation Program (award NSF CHE-1827457) and Vagelos Institute for Energy Science and Technology used in this study
3	Origin	Bruker BioSpin GmbH
4	Owner	nmrsu
5	Site	
6	Instrument	Avance
7	Author	
8	Solvent	CDCl3
9	Temperature	298.0
10	Pulse Sequence	zgpg30
11	Experiment	1D
12	Probe	Z152088_0015 (PI HR-400-S1-BBF/ H/ D-5.0-Z SP N)
13	Number of Scans	3072
14	Receiver Gain	15.6
15	Relaxation Delay	2.0000
16	Pulse Width	9.0500
17	Presaturation Frequency	
18	Acquisition Time	1.3763
19	Acquisition Date	2021-08-11T02:23:14
20	Spectrometer Frequency	100.63
21	Spectral Width	23809.5
22	Lowest Frequency	-1832.3
23	Nucleus	13C
24	Acquired Size	32768
25	Spectral Size	65536

(3R,5S)-1-allyl-5-((5-(3-fluoropropyl)-2,3-dimethoxybenzamido)methyl)pyrrolidin-3-yl (8-(thiophen-3-yl)octyl)carbamate (***(R,S)-trans-2j***). GT-7-15

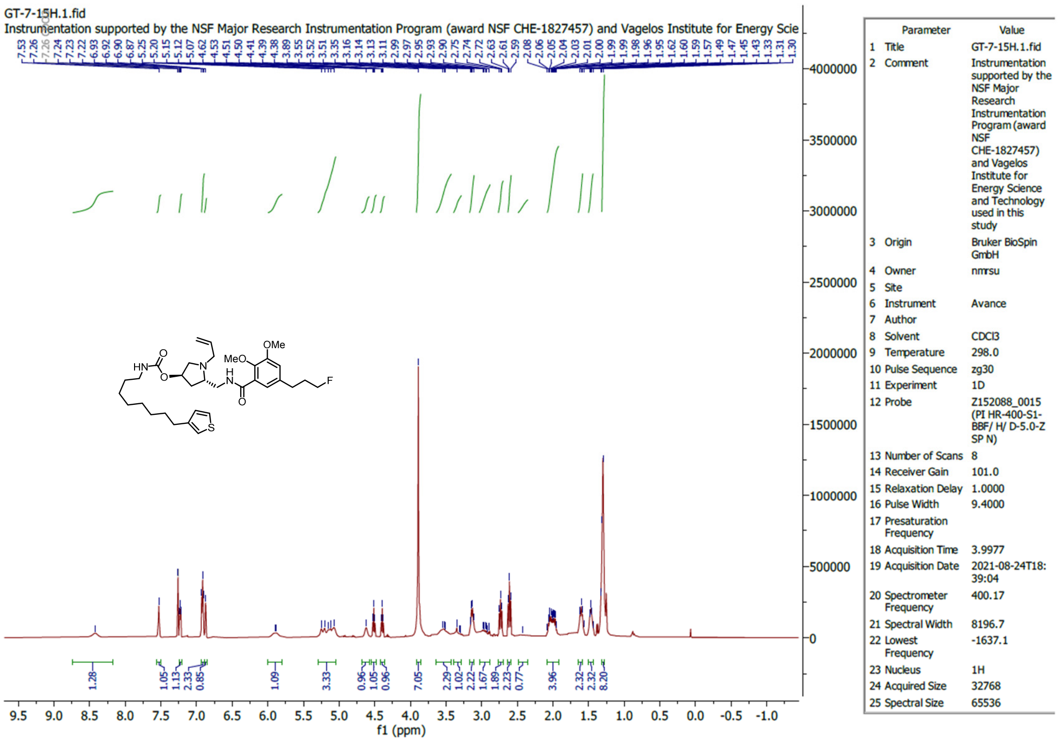

Parameter	Value
1 Title	GT-7-15H.1.fid
2 Comment	Instrumentation supported by the NSF Major Research Instrumentation Program (award NSF CHE-1827457) and Vagelos Institute for Energy Science and Technology used in this study
3 Origin	Bruker BioSpin GmbH
4 Owner	nmrsu
5 Site	
6 Instrument	Avance
7 Author	
8 Solvent	CDCl3
9 Temperature	298.0
10 Pulse Sequence	zg30
11 Experiment	1D
12 Probe	Z152088_0015 (PI HR-400-S1-BBF/ H/ D-5.0-Z SP N)
13 Number of Scans	8
14 Receiver Gain	101.0
15 Relaxation Delay	1.0000
16 Pulse Width	9.4000
17 Presaturation Frequency	
18 Acquisition Time	3.9977
19 Acquisition Date	2021-08-24T18:39:04
20 Spectrometer Frequency	400.17
21 Spectral Width	8196.7
22 Lowest Frequency	-1637.1
23 Nucleus	1H
24 Acquired Size	32768
25 Spectral Size	65536

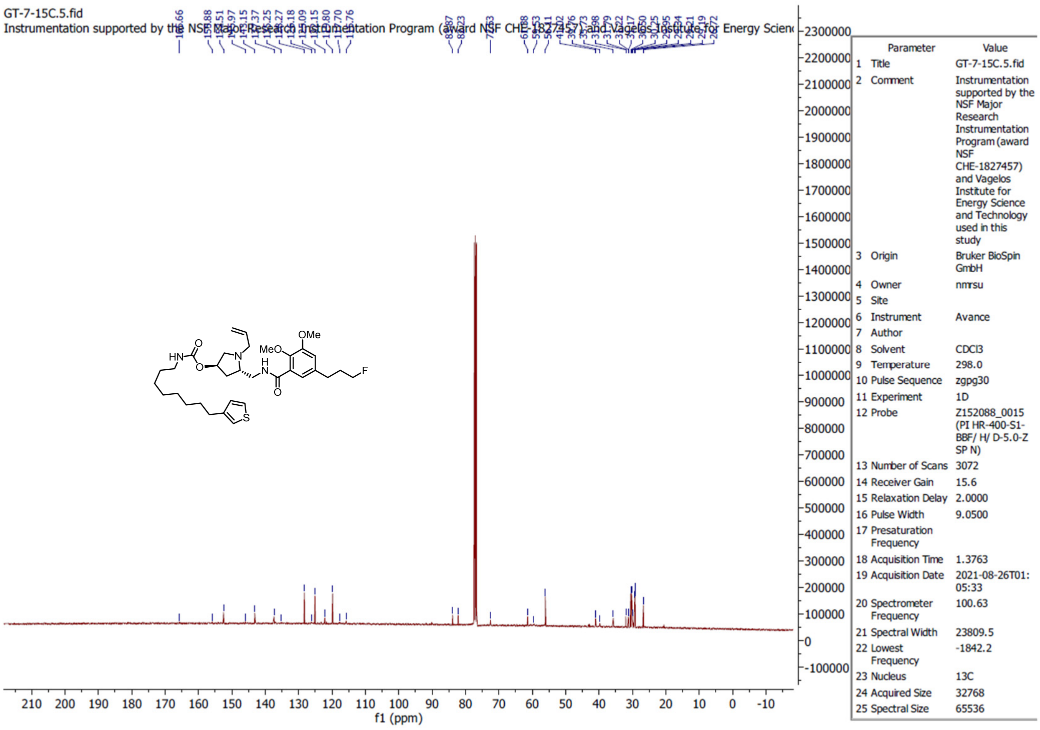

Parameter	Value
1 Title	GT-7-15C.5.fid
2 Comment	Instrumentation supported by the NSF Major Research Instrumentation Program (award NSF CHE-1827457) and Vagelos Institute for Energy Science and Technology used in this study
3 Origin	Bruker BioSpin GmbH
4 Owner	nmrsu
5 Site	
6 Instrument	Avance
7 Author	
8 Solvent	CDCl3
9 Temperature	298.0
10 Pulse Sequence	zgpg30
11 Experiment	1D
12 Probe	Z152088_0015 (PI HR-400-S1-BBF/ H/ D-5.0-Z SP N)
13 Number of Scans	3072
14 Receiver Gain	15.6
15 Relaxation Delay	2.0000
16 Pulse Width	9.0500
17 Presaturation Frequency	
18 Acquisition Time	1.3763
19 Acquisition Date	2021-08-26T01:05:33
20 Spectrometer Frequency	100.63
21 Spectral Width	23809.5
22 Lowest Frequency	-1842.2
23 Nucleus	13C
24 Acquired Size	32768
25 Spectral Size	65536

(3S,5S)-1-allyl-5-((5-(3-fluoropropyl)-2,3-dimethoxybenzamido)methyl)pyrrolidin-3-yl decylcarbamate (***(S, S)-cis-2d***) GT-7-52 *and (3R,5R)-1-allyl-5-((5-(3-fluoropropyl)-2,3-dimethoxybenzamido)methyl)pyrrolidin-3-yl decylcarbamate* (***(R, R)-cis-2d***). GT-7-54

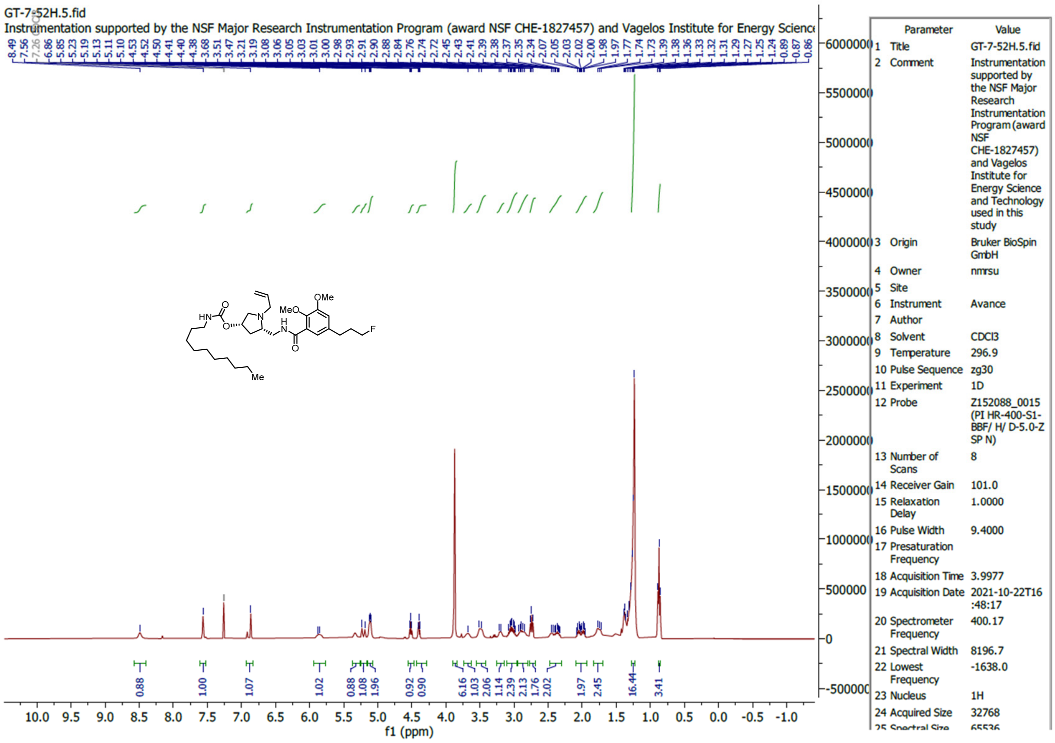

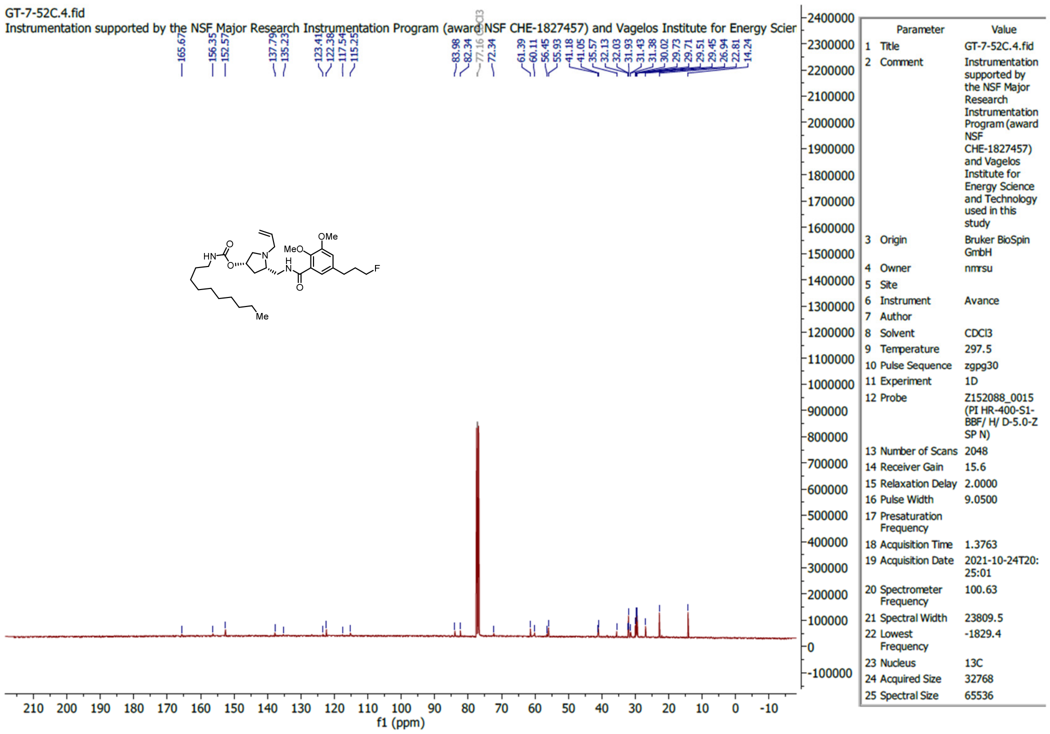

(3S,5S)-1-allyl-5-((5-(3-fluoropropyl)-2,3-dimethoxybenzamido)methyl)pyrrolidin-3-yl (8-(thiophen-2-yl)octyl)carbamate (***(S, S)-cis-2i***) GT-7-53 *and (3R,5R)-1-allyl-5-((5-(3-fluoropropyl)-2,3-dimethoxybenzamido)methyl)pyrrolidin-3-yl (8-(thiophen-2-yl)octyl)carbamate* (***(R, R)-cis-2i***). GT-7-55

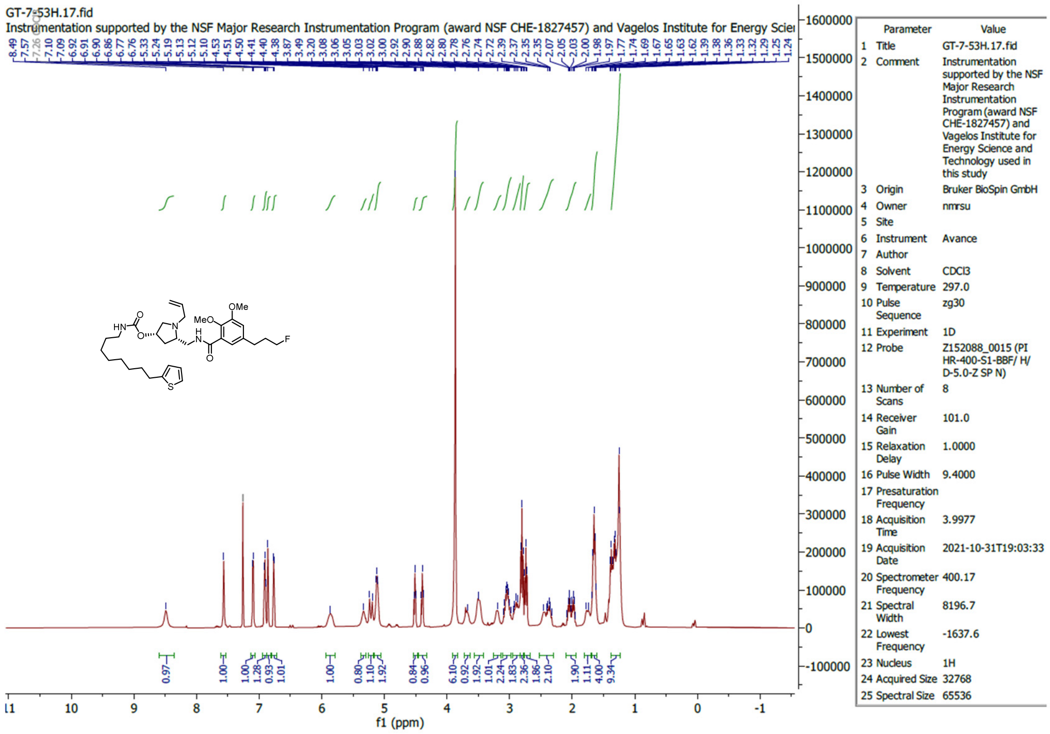

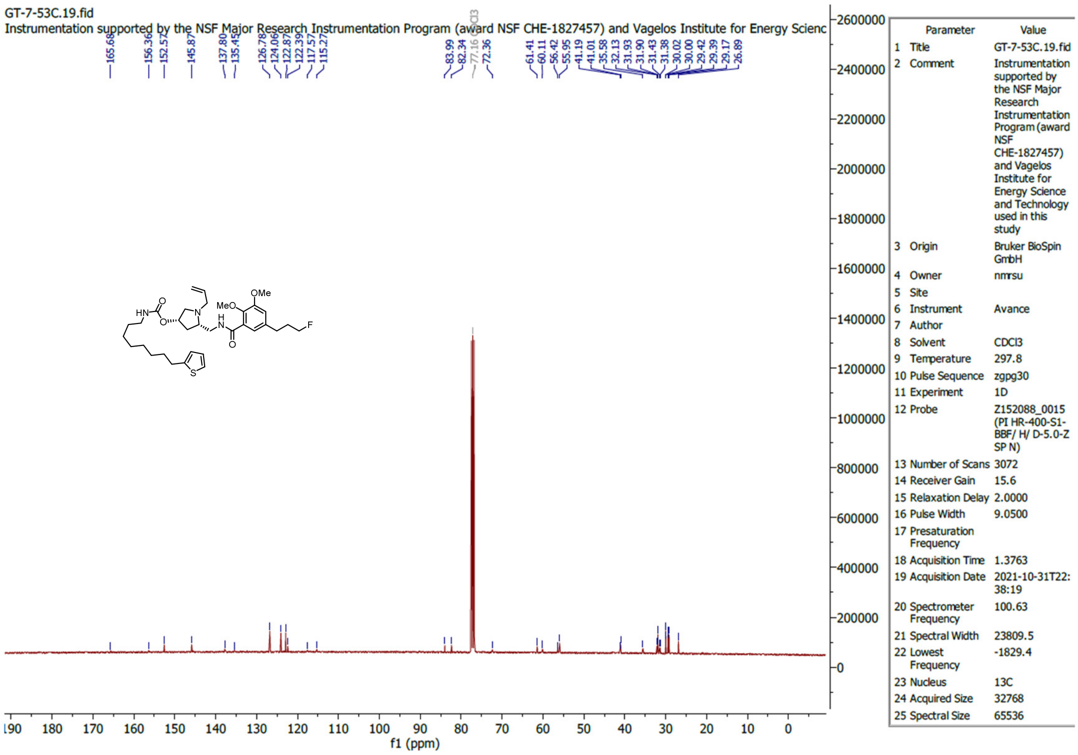

Supplement: Supplementary file 1 [file molecules-29-00123-s001.zip › molecules-2711893-supplementary.pdf]
